# Utilization of ethanolamine phosphate phospholyase as a unique astrocytic marker

**DOI:** 10.3389/fncel.2023.1097512

**Published:** 2023-01-30

**Authors:** Hiroshi Tsujioka, Toshihide Yamashita

**Affiliations:** ^1^Graduate School of Medicine, Osaka University, Osaka, Japan; ^2^WPI Immunology Frontier Research Center, Osaka University, Osaka, Japan; ^3^Graduate School of Frontier Biosciences, Osaka University, Osaka, Japan; ^4^Department of Neuro-Medical Science, Graduate School of Medicine, Osaka University, Osaka, Japan

**Keywords:** *Etnppl*, *Agxt2l1*, astrocyte, marker, developmental change, pyramidotomy, monoclonal antibody, *Gjb6*

## Abstract

Astrocytes play diverse roles in the central nervous system (CNS) in both physiological and pathological conditions. Previous studies have identified many markers of astrocytes to analyze their complicated roles. Recently, closure of the critical period by mature astrocytes has been revealed, and the need for finding mature astrocyte-specific markers has been growing. We previously found that *Ethanolamine phosphate phospholyase* (*Etnppl*) was almost not expressed in the developing neonatal spinal cord, and its expression level slightly decreased after pyramidotomy in adult mice, which showed weak axonal sprouting, suggesting that its expression level negatively correlates with axonal elongation. Although the expression of *Etnppl* in astrocytes in adult is known, its utility as an astrocytic marker has not yet been investigated in detail. Here, we showed *that Etnppl* was selectively expressed in astrocytes in adult. Re-analyses using published RNA-sequencing datasets revealed changes in *Etnppl* expression in spinal cord injury, stroke, or systemic inflammation models. We produced high-quality monoclonal antibodies against ETNPPL and characterized ETNPPL localization in neonatal and adult mice. Expression of ETNPPL was very weak in neonatal mice, except in the ventricular and subventricular zones, and it was heterogeneously expressed in adult mice, with the highest expression in the cerebellum, olfactory bulb, and hypothalamus and the lowest in white matter. Subcellular localization of ETNPPL was dominant in the nuclei with weak expression in the cytosol in the minor population. Using the antibody, astrocytes in adult were selectively labeled in the cerebral cortex or spinal cord, and changes in astrocytes were detected in the spinal cord after pyramidotomy. ETNPPL is expressed in a subset of *Gjb6*^+^ astrocytes in the spinal cord. The monoclonal antibodies we created, as well as fundamental knowledge characterized in this study, will be valuable resources in the scientific community and will expand our understanding of astrocytes and their complicated responses in many pathological conditions in future analyses.

## 1. Introduction

Astrocytes make up a major cell population in the central nervous system (CNS) and account for 20–50% of cells in the CNS (Hasel and Liddelow, 2021). They play diverse roles in the CNS and are indispensable for normal brain function. For example, the endfeet of astrocytes contact the pre- and post-synapses of a subset of neurons forming a functional unit called a tripartite synapse, in which astrocytes are activated by neurotransmitters, integrate information, and release gliotransmitters Ca^2+^ dependently to regulate synaptic transmission (Perea et al., 2009). Their endfeet also ensheath endothelial cells, pericytes, and basement membrane and regulate blood flow depending on neuronal activity. They also regulate the expression of junctional proteins by endothelial cells, leading to the prevention of diffusion of macromolecules into the brain parenchyma as an important component of the blood-brain barrier (Daneman and Prat, 2015). Astrocytes also phagocytose certain components, especially synapses during development, to shape an appropriate neural network (Konishi et al., 2022). Astrocytes play important roles not only in physiological conditions but also in pathological conditions. For example, they show hypertrophic morphology and surround the lesion, comprising glial scar after injury, which restricts inflammation whereas it also inhibits axonal regeneration as a physical and chemical barrier (Sofroniew, 2014). Under some conditions, neurotoxic lipids are secreted from astrocytes to induce neuronal death (Guttenplan et al., 2021). They also secrete both pro- and anti-inflammatory factors that modulate inflammation (Sofroniew, 2015).

Identifying specific markers and developing useful tools are crucial for understanding such versatile and important astrocytic functions. Many astrocytic markers have been identified in previous studies (Jurga et al., 2021). For example, glial fibrillary acidic protein (GFAP) is the most widely used astrocytic marker (Eng et al., 1971). It is a cytoskeletal protein, and the morphology of astrocytes is clearly visualized using an anti-GFAP antibody. Because it is mainly expressed in white matter astrocytes, in many cases, other markers such as S100 protein beta (S100B) (Boyes et al., 1986) are used to label gray matter astrocytes. To label all astrocytes, a transgenic mouse line for the *aldehyde dehydrogenase 1 family member L1* (*Aldh1l1*) is widely used (Yang et al., 2011). Since recent single-cell RNA-sequencing (scRNA-seq) studies have revealed high heterogeneity of astrocytes (Batiuk et al., 2020; Bayraktar et al., 2020; Hasel et al., 2021; Shi et al., 2021), the demand for adding new astrocytic markers and tools to analyze heterogeneous and complicated astrocytic roles is now growing.

Among the complicated astrocytic functions, the regulation of neuronal plasticity by postnatally mature astrocytes is becoming an interesting topic. If neonatal astrocytes are transplanted into the visual cortex of adult cats, the critical period reopens and ocular dominance changes after monocular closure (Müller and Best, 1989), indicating that mature astrocytes regulate neuronal plasticity. Recently, the molecular mechanism of this phenomenon has been revealed, and molecules such as *gap junction protein beta 6* (*Gjb6*), which is specifically expressed in mature astrocytes, have been shown to be responsible for the closure of the critical period in mice (Ribot et al., 2021). Astrocytes are also essential for the closure of the critical period of the motor circuit of *Drosophila* (Ackerman et al., 2021), indicating that the importance of astrocyte maturation for neuronal plasticity is not limited to the visual system.

Previously, to elucidate the mechanisms underlying the high ability to restore damaged neural function in neonatal mice, we compared gene expression profiles in the cervical cord between neonatal mice and adult mice, which showed prominent axonal sprouting and limited axonal sprouting, respectively, after corticospinal tract (CST) injury (pyramidotomy) (Tsujioka and Yamashita, 2019). We found that *ethanolamine phosphate phospholyase* (*Etnppl*) showed the most prominent difference between intact neonates and adults. It is almost not expressed in neonates, and its expression level decreases slightly after pyramidotomy in adult mice, suggesting that its expression level negatively correlates with axonal elongation. Interestingly, previous studies have shown that it is expressed in astrocytes in humans (Leventoux et al., 2020) and mice (White et al., 2021). Although these findings suggest the potential of *Etnppl* as a mature astrocytic marker, very few studies have focused on *Etnppl*, and useful tools, such as monoclonal antibodies, have not been developed.

In this study, we investigated *Etnppl* expression. Cell sorting followed by quantification of mRNA levels revealed that *Etnppl* is selectively expressed in astrocytes in adult. Re-analysis of previously published RNA-seq datasets revealed that the expression level of *Etnppl* was downregulated or upregulated depending on the type of stimulation in several pathological conditions. We created high-quality anti-ETNPPL monoclonal antibodies and validated their specificity using knockout mice. Using this antibody, we revealed that the expression of ETNPPL was very weak in neonates, and it was heterogeneously expressed in adults with nuclear-dominant subcellular localization. We confirmed the antibody labels astrocytes selectively using IHC. We showed that the monoclonal antibody detected a decrease in ETNPPL^+^ astrocytes after pyramidotomy. ETNPPL^+^ cells were a subset of *Gjb6*^+^ astrocytes. These results show the usefulness of the anti-ETNPPL monoclonal antibody we produced and the potential importance of ETNPPL as a marker of astrocytes in adult, which might reflect unique astrocytic changes in pathological conditions.

## 2. Materials and methods

### 2.1. Animals

C57BL/6J wild-type (WT) mice were purchased from Japan SLC. In our attempt to obtain *Etnppl*-floxed mice (C57BL/6J background) using the clustered regularly interspaced short palindromic repeat/CRISPR-associated 9 system (Miyasaka et al., 2018), we obtained *Etnppl* knockout (KO) mice and used them in this study. All mice used in this study were female. Mice were maintained under specific pathogen-free 12 h light/12 h dark cycle conditions and fed *ad libitum*.

### 2.2. Surgery

Pyramidotomy was performed as previously described (Starkey et al., 2005; Tsujioka and Yamashita, 2019). In brief, mice were completely anesthetized with an intraperitoneal injection of a three-drug mix (medetomidine, midazolam, and butorphanol) or inhalation of isoflurane. The ventral side of the neck was incised, the esophagus and trachea were retracted, and the medullary pyramid on the left side of the basal artery was injured approximately 0.25 mm in depth and 0.5 mm in width by sharp knives. For the sham group, the pyramid was not injured, and the skin incision was closed.

### 2.3. Cell sorting

Magnetic activated cell sorting (MACS) was performed using Adult Brain Dissociation Kit (Miltenyi, 130-107-677) following the manufacturer’s protocol. In brief, the spinal cords of 3–5 mice, which correspond approximately to the C4-L2 vertebral level, were pooled for each sample, minced, and dissociated in enzyme P and A using gentleMACS C tubes (Miltenyi, 130-096-334) and gentleMACS Octo Dissociator with Heaters (Miltenyi, 130-096-427). Debris was removed using a debris removal solution. The cell suspension was stained with 3 μg/ml of anti-astrocyte cell surface antigen 2 (ACSA2)-PE (Miltenyi, 130-116-244) and 1 μg/ml of 4′,6-diamidino-2-phenylindole (DAPI) for 10 min at 4°C. After washing, the cell suspension was incubated with 20% volume of anti-PE MicroBeads UltraPure (Miltenyi, 130-105-639) for 15 min at 4°C. Aliquots were stored and used as the original cell suspensions for quality check analysis. Bead-labeled cells were trapped in LS columns (Miltenyi, 130-042-401) under a magnetic field of QuadroMACS Separator (Miltenyi, 130-090-976). Two LS columns were used for each sample for purification. Flow-through (FT) of LS columns (FT fraction) or elution fraction (ACSA2^+^ fraction) was used for expression analysis in the following section, or aliquots were used for quality-check analysis.

For quality-check analysis by fluorescence-activated cell sorting (FACS), the cell suspension was passed through a 100 μm mesh and analyzed using FACS Verse (BD).

### 2.4. Quantitative reverse transcription-polymerase chain reaction (qRT-pCR)

**FIGURE 1:**
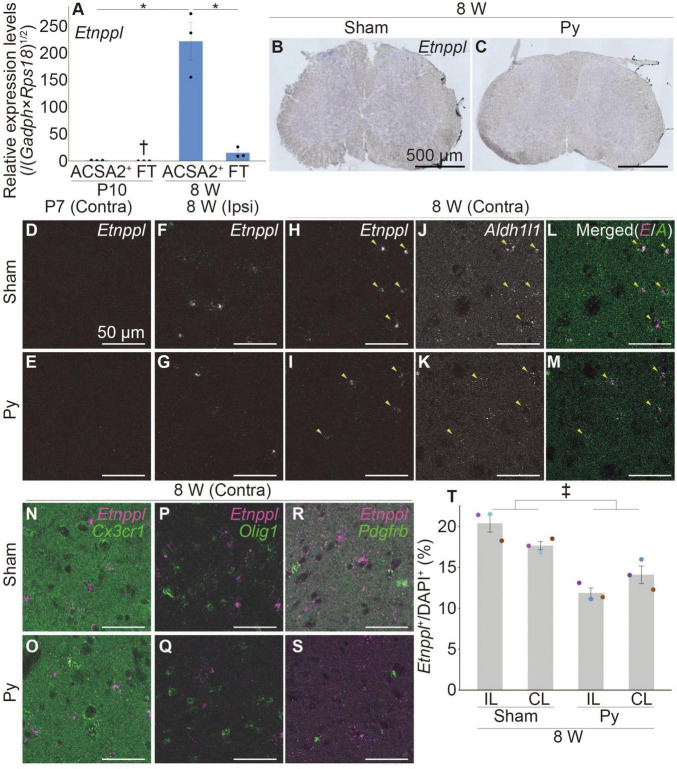
*Etnppl*-expression in astrocytes in adult and its expression change after pyramidotomy. **(A)** *Etnppl* expression in MACS-isolated ACSA2^+^ astrocytes. The vertical axis represents the relative expression level of *Etnppl* normalized by the geometric mean of *glyceraldehyde-3-phosphate dehydrogenase* (*Gapdh*) and *ribosomal protein S18* (*Rps18*), taking the value of the ACSA2^+^ P10 sample as one. Mean ± S.E.M. *n* = 3, *P < 0.05, Tukey’s HSD test; ^†^below the limit of quantification. FT, flow-through. **(B,C)** Localization of *Etnppl*, as revealed by ISH. Cervical cord of 8 W female mice 3 days after sham surgery **(B)** or pyramidotomy **(C)** are shown. *The Etnppl* signal is indicated by blue and purple. The right side is marked with black pigment. Dorsal is top, left is to the left. Scale bars: 500 μm. **(D–S)** Localization of *Etnppl* and other cell markers revealed by RNAscope. Confocal enlarged images of gray matter of the contralateral **(D,E,H–S)** or ipsilateral **(F,G)** side of the cervical cord of female mice that were subjected to sham surgery **(D,F,H,J,L,N,P,R)** or pyramidotomy **(E,G,I,K,M,O,Q,S)** at P7 **(D,E)** or 8 W **(F–S)** 3 days before fixation. White indicates the signal for *Etnppl* **(D–I)** or *Aldh1l1* **(J,K)**. Magenta in the merged images **(L–S)** indicates the *Etnppl* signal. Green indicates signals for *Aldh1l1* **(L,M)**, *Cx3cr1* **(N,O)**, *Olig1* **(P,Q)**, or *Pdgfrb* **(R,S)**. Yellow arrowheads indicate *Etnppl*-*Aldh1l1* double positive cells. Scale bars: 50 μm. **(T)** Quantification of *Etnppl*^+^ cells. The vertical axis represents the percentage of *Etnppl*^+^ cells among all cells (DAPI^+^). Mean ± S.E.M. *n* = 3 individuals, each of which is an average of 3–4 different sections. ‡*P* < 0.05, Sham vs. Py; *P* < 0.05, interaction effect; two-way ANOVA; IL, ipsilateral; CL, contralateral. The same color indicates the same individual.

qRT-PCR in Figure 1 was performed as described previously (Tsujioka and Yamashita, 2019). In brief, RNA was extracted using RNeasy mini kit (Qiagen, 74104) with RNase-Free DNase Set (Qiagen, 79254) from cells obtained from the “Cell sorting” section.

**FIGURE 2:**
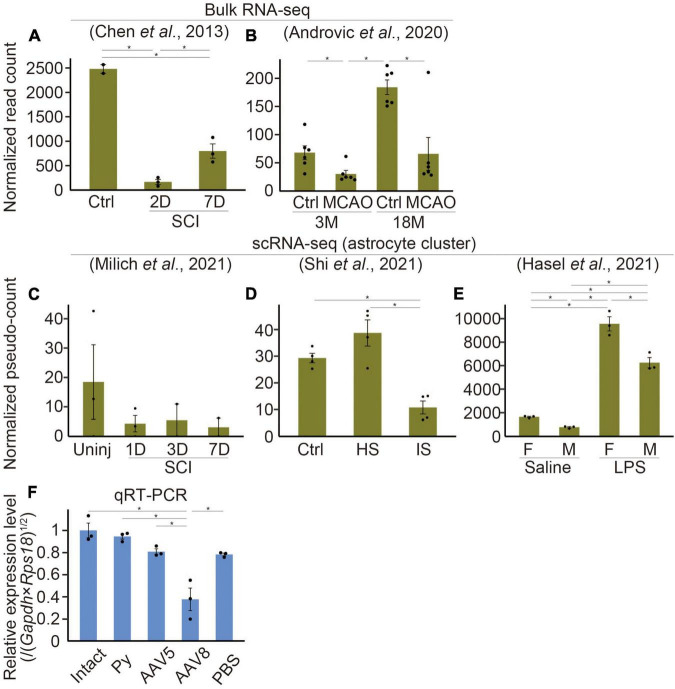
*Etnppl*-expression in several kinds of diseases. **(A–E)** Expression levels of *Etnppl* in bulk RNA-seq **(A,B)** or scRNA-seq **(C–E)** datasets. Vertical axes represent normalized read counts of *Etnppl* in the tissue **(A,B)** or normalized pseudo-counts in the astrocyte cluster **(C–E)**. *P < 0.05, Wald test. Ctrl, control; D, days after injury; 3M, 3 months of age; Uninj, uninjured; HS, hemorrhagic stroke; IS, ischemic stroke; F, female; M, male. **(F)** Expression level of *Etnppl* in the cervical cord 2 weeks after AAV infection as revealed by qRT-PCR. The vertical axis represents the relative expression level of *Etnppl* normalized by the geometric mean of *Gapdh* and *Rps18*, taking the value of the intact group as one. *P < 0.05, Tukey’s HSD test. Mean ± S.E.M. *n* is equal to the number of dots in each panel.

For expression analysis of the spinal cord tissue in Figure 2, 0.2 μl of 1 × 10^13^ genome copy/ml of enhanced green fluorescent protein (EGFP)-expressing adeno-associated virus (AAV) vector serotype 5 or 8 (VectorBuilder, 3AAVSP01-5, 8) or phosphate buffered saline (PBS) were injected into 6 points of the cervical cord (C4 and C6 vertebral levels, 0.4 mm right from the midline, depth = 0.8, 0.5, and 0.3 mm; total 1.2 μl) using NANOLITER2010 (World Precision Instruments) at a rate of 100 nl/min, or pyramidotomy was performed in non-injected mice. Two weeks after surgery, cervical cord tissues corresponding to C4–7 vertebral levels were harvested, and RNA was extracted as described above.

Total RNA was reverse-transcribed using a High Capacity cDNA Reverse Transcription Kit (Applied Biosystems, 4368814). Quantitative PCR was performed using the Fast SYBR Green Master Mix (Applied Biosystems, 4385612) and QuantStudio 7 Flex Real-Time PCR System (Applied Biosystems). Relative expression levels were calculated by the relative standard curve method using serial dilutions of cDNA as a standard, and if the expression level was below the log-linear range of the standard, it was regarded as below the limit of quantification. For statistical analysis, Tukey’s honestly significant difference (HSD) test was used after removing samples below the limit of quantification, with a cut-off of *P* < 0.05. The primers used for qRT-PCR are listed in Table 1.

**TABLE 1:** Primers used for qRT-PCR.

Gene	Forward primer	Reverse primer
*Etnppl*	5′-gccaatgacttagccttacg-3′	5′-taagcatggtcaagggtgat-3′
*Gapdh*	5′-tgtgtccgtcgtggatctga-3′	5′-ttgctgttgaagtcgcaggag-3′
*Rps18*	5′-catgcagaacccacgacagta-3′	5′-cctcacgcagcttgttgtcta-3′

### 2.5. Histological analyses

Histological analyses were performed as described previously (Tsujioka and Yamashita, 2019), with modifications. In brief, brains were harvested after perfusion with 4% paraformaldehyde (PFA) in PBS followed by post-fixation in 4% PFA/PBS for 3 overnights (o/n) for *in situ* hybridization (ISH), 24 h for RNAscope, or 6 h for immunohistochemistry (IHC), and tissue was frozen. Spinal cord tissues corresponding to approximately the C7 vertebral level (∼C7–8 spinal cord segments), T6 vertebral level (∼T7–8 spinal cord segments), or T12 vertebral level (∼L2–3 spinal cord segments Harrison et al., 2013) were used as the cervical, thoracic, or lumbar cord tissues, respectively.

For ISH, 20 μm-thickness sections were floated on PBS, treated with 0.2 μg/ml of proteinase K for 30 min at 37°C, acetylated, hybridized with digoxigenin (DIG)-labeled *Etnppl* probe, treated with RNase, treated with alkaline phosphatase-conjugated anti-DIG antibody, incubated with nitro blue tetrazolium chloride/5-bromo-4-chloro-3-indolyl-phosphate, toluidine-salt solution, and washed with methanol.

The RNAscope assay (Wang et al., 2012) was performed using the RNAscope Multiplex Fluorescent Kit v2 (Advanced Cell Diagnostics, 323100) following the manufacturer’s protocol. Sixteen μm-thickness sections were mounted on glass slides, treated with 4% PFA, treated with hydrogen peroxide, boiled in target retrieval reagent for 5 min, treated with protease III, hybridized with RNAscope probe [Advanced Cell Diagnostics, *Etnppl*: 825341, *Aldh1l1*: 405891-C2, *chemokine (C-X3-C motif) receptor 1* (*Cx3cr1)*: 314221-C3, *oligodendrocyte transcription factor 1* (*Olig1)*: 480651-C3, *platelet derived growth factor receptor, beta polypeptide (Pdgfrb)*: 411381-C4, *Gjb6*: 458811, *angiotensinogen (serpin peptidase inhibitor, clade A, member 8)* (*Agt)*: 426941, *inter-alpha trypsin inhibitor, heavy chain 3* (*Itih3)*: 840071], incubated with Opal 690, 620, or 520 (PerkinElmer, FP1497001KT, FP1495001KT, FP1487001KT), and treated with DAPI.

For IHC for protein kinase C gamma (PKCγ), 20 μm-thickness sections were mounted on glass slides, incubated with 400 ng/ml of anti-PKCγ antibody (Santa Cruz Biotechnologies, sc-211), and treated with 4 μg/ml of Alexa Fluor 568-conjugated anti-rabbit IgG antibody (Invitrogen, A11011). For IHC for other proteins, 10 μm-thickness sections were mounted on glass slide, incubated with 1/250 dilution of anti-ETNPPL monoclonal antibodies (or 1/50 for screening), 100 ng/ml of anti-S100B antibody (Abcam, ab52642), 125 ng/ml of anti-neuronal nuclei (NeuN) antibody (Millipore, MAB377), 500 ng/ml of anti-ionized calcium-binding adapter molecule 1 (IBA1) antibody (Wako, 019-19741), 1/1,000 dilution of anti-glutathione S-transferase pi (GSTπ) antibody (MBL, 312), 5 μg/ml of anti-neuron-glial antigen 2 (NG2) antibody (Millipore, AB5320), 670 ng/ml of anti-PDGFRA antibody (Cell Signaling, 3174T), 500 ng/ml of anti-OLIG2 antibody (Millipore, MABN50), 1/1,000 dilution of chicken anti-GFAP antibody (Abcam, ab4674), ready-to-use rabbit anti-GFAP antibody (Dako, IR524), or 1/200 dilution of goat anti-SRY (sex determining region Y)-box 9 (SOX9) antibody (R&D, AF3075) followed by incubation with 4 μg/ml of Alexa Fluor 647, 568, or 488-conjugated anti-rat, -rabbit, -mouse, or -goat IgG antibody (Invitrogen) or 2 μg/ml of Alexa Fluor 488 conjugated anti-chicken IgY antibody (Abcam, ab150169). For the combination of RNAscope and IHC, the primary antibody for IHC was applied after Opal treatment of the RNAscope assay, followed by incubation with the secondary antibody. One μg/ml DAPI was applied with secondary antibodies.

### 2.6. Imaging and quantification of histological samples

Images were acquired using fluorescence microscopes or a confocal laser scanning microscope FV3000 (Olympus). If a confocal microscope was used, this is described in the figure legends. For quantification, a 300 μm × 300 μm region of interest (ROI) was set in each section, and the number of marker-positive cells was counted manually. Since the fluorescence intensity of the signal and background varies between sections, we did not consider intensity, and we included all positive cells ranging from weakly positive to strongly positive as positive. An average of 3–4 different sections of the same individual was considered as the value of the individual, and using three individuals for each group, statistical analysis was performed (*n* = 3). Two-way analysis of variance (ANOVA) was performed to test differences between conditions (sham vs. Py) and differences between laterality (ipsilateral vs. contralateral), with a cut-off of *P* < 0.05. For quantification of the number of dots in RNAscope assay, we essentially followed the namufacturer’s protocol. In brief, four *Gjb6* negative areas (30–50 μm × 30–50 μm each) or 20 single dots for *Gjb6* signal were chosen for quantification of background or single dot intensity, respectively, in each image. After subtracting background intensity, intensity for single dot was calculated, total intensity was divided by the intensity for single dot, and the value was shown as dots per 1 mm^2^. The following procedure is the same as described above.

### 2.7. RNA-seq data analysis

Data processing for bulk RNA-seq was performed as previously described (Tsujioka and Yamashita, 2019), with modifications. In brief, raw read data for the spinal cord injury (SCI) model (Chen et al., 2013) or middle cerebral artery occlusion (MCAO) stroke model (Androvic et al., 2020) were downloaded from the sequence read archive (SRA; SRP019916 or SRP221729, respectively). Reads were quality-checked, trimmed using fastp (Chen et al., 2018), and mapped to the mouse genome GRCm39 (Cunningham et al., 2022) using STAR (Dobin et al., 2013). The read count was calculated using HTSeq (Anders et al., 2015) with Mus_musculus.GRCm39.104.gtf (Cunningham et al., 2022) as a reference gene model, and statistical analysis was performed using the DESeq2 (Love et al., 2014) package in R (R Core Team, 2022). The Wald test was used for statistical analysis, with a cut-off of adjusted *P* < 0.05. Genes were annotated using org.Mm.eg.db (Carlson, 2018), and normalized read counts were plotted using ggplot2 (Wickham, 2009) and ggbeeswarm (Clarke and Sherrill-Mix, 2017).

Data processing for scRNA-seq was performed as previously described (Ikeda-Yorifuji et al., 2022), with modifications. Raw read data for the stroke model (Shi et al., 2021) were downloaded from the SRA (SRP308347). The count matrix was retrieved using 10x Genomics Cell Ranger 3.1.0 (Zheng et al., 2017). Alternatively, the count matrix of the systemic inflammation model (Hasel et al., 2021) was downloaded from Gene Expression Omnibus (GEO; GSE148611). Quality checks were performed using the Seurat (Hao et al., 2021) package of R with the following cutoffs: nCount < ± 2 S.D., nFeature < ± 2 S.D., and percent mitochondrial gene < 2 S.D. SCTransform v2 was applied and samples were integrated. The number of principal components used for the following analyses was determined using findPC (Zhuang et al., 2022) with the parameters set as the first derivative, 50 PCs, and clustering was performed with a default resolution of 0.8. Reference-based automated annotation was performed using SingleR (Aran et al., 2019) with celldex:MouseRNAseqData as the reference. Alternatively, the annotated count matrix of the SCI model (Milich et al., 2021) was downloaded from the GEO (GSE162610). The specificity of cell markers was visualized using the DotPlot function of Seurat. The astrocyte cluster was selected, and pseudobulk analysis using DESeq2 was performed as previously described (Mary et al., 2022). In brief, counts of astrocyte clusters in each sample were aggregated, and statistical tests and plotting were performed as described above.

### 2.8. Plasmid construction and antibody production

Plasmid construction was performed as described previously (Tsujioka et al., 2017), with modifications. Briefly, the coding sequence (CDS) with part of the untranslated region (UTR) of *Etnppl* was cloned into the pGEM-T Easy Vector (Promega, A1360) to produce the etnppl-pGEMtEasy vector. The *Etnppl* sequence was subcloned into the *Nde*I restriction enzyme recognition site of pET-15b (Millipore, 69661-3CN) or *Eco*RI or *Not*I recognition site of pSF-CMV-NEO-NH2-3XFLAG (Sigma, OGS627-5UG) using the In-Fusion HD Cloning Kit (Clontech, 639648), which produced pET-15b(ETNPPL), p3xFLAG-Etnppl, or pEtnppl-3xFLAG plasmids, respectively. etnppl-pGEMtEasy was used to produce the ISH probes. pET-15b(ETNPPL) was used to produce His-tagged recombinant ETNPPL as an antigen for monoclonal antibody production. A monoclonal antibody producing service by Immuno-Biological Laboratories was used to produce the antibodies. In brief, His-tagged recombinant ETNPPL was purified by Ni-NTA column, and four rats were immunized. Their serum were screened and best candidate was chosen. The lymphocytes were fused with X-63/Ag8.653 myeloma. The hybridoma were cloned by limiting dilution. Then candidate clones were screened. p3xFLAG-Etnppl and pEtnppl-3xFLAG were used to prepare the input samples for immunoprecipitation.

### 2.9. Immunoprecipitation (IP)

The KT-5 mouse astrocyte cell line (Satoh et al., 1999) provided by RIKEN BioResource Center (BRC; IFO50161) was transfected with p3xFLAG-Etnppl or pEtnppl-3xFLAG using SG Cell Line 4D-Nucleofector X Kit L (Lonza, V4XC-3024) and 4D-Nucleofector system (Lonza) following the manufacturer’s protocol. IP was performed using Dynabeads Protein G for Immunoprecipitation (Invitrogen, 10004D) and DynaMag-2 (Invitrogen, 12321D), following the manufacturer’s protocol. In brief, 10 μg/ml of anti-ETNPPL monoclonal antibody or normal rat serum IgG (Sigma, I4131-10MG) was incubated with lysate o/n at 4°C, followed by incubation with dynabeads for 1 h at 4°C. After washing the beads, immunoprecipitated proteins were eluted by direct boiling of beads in SDS-PAGE sample buffer (58 mM Tris pH 6.8, 2% sodium dodecyl sulfate, 10% glycerol, 0.005% bromophenol blue, 3% β-mercaptoethanol).

### 2.10. Western blotting (WB)

Cervical cord tissues corresponding to C4–7 vertebral levels of 8-week old (8W) female *Etnppl*^–/–^ or *Etnppl*^±^ mice were homogenized in RIPA buffer [50 mM Tris (pH 8.0), 150 mM NaCl, 1% NP-40, 0.5% sodium deoxycholate, 0.1% sodium dodecyl sulfate] with cOmplete EDTA free (Roche, 11873580001) using Micro Smash (Tomy). The total protein concentration was measured using the Pierce BCA Protein Assay Kit (Pierce, 23227) following the manufacturer’s protocol.

Western blotting was performed using a Mini Protean tetra cell (BioRad, 1658005JA) and Mini Trans-Blot module (BioRad, 1703935JA), following the manufacturer’s protocol. In brief, 7 μg of total protein was applied to 12% Mini Protean TGX Stain-free pre-cast gel (BioRad, 4568046). Electrophoresis was performed, Stain-free was activated, protein on the gel was blotted on 0.2 μm pore size nitrocellulose membrane, the membrane was incubated with 1/100 dilution of the monoclonal antibodies, followed by incubation with 1/3,000 dilution of horseradish peroxidase-conjugated anti-rat IgG antibody (Cell Signaling, 7077). After acquiring the Stain-free signal, Pierce ECL Plus Western Blotting Substrate (Pierce, 32132) was added, and the signal for ETNPPL was acquired. For WB using IP samples, immunoprecipitated samples or input samples corresponding to 10% amount of immunoprecipitated sample were applied on 4–20% Mini Protean TGX pre-cast gels (BioRad, 4501096). Primary and secondary antibodies, 1 μg/ml of anti-FLAG antibody (Sigma, F1804) and 1/5,000 dilution of StarBright Blue 700-conjugated anti-mouse IgG antibody (BioRad, 12004158) were used, respectively.

## 3. Results

To examine whether *Etnppl* is expressed in astrocytes in adult, we first isolated cells expressing the astrocytic marker astrocyte cell surface antigen 2 (ACSA2) (Kantzer et al., 2017) from the neonate (postnatal day 10; P10) and adult (8 weeks age; 8 W) spinal cords using a magnetic associated cell sorter (MACS). We obtained highly purified ACSA2^+^ cells (approximately 90%, Supplementary Figure 1) and quantified their *Etnppl*-mRNA levels by quantitative reverse transcription-polymerase chain reaction (qRT-PCR). *Etnppl* was almost exclusively expressed in adult ACSA2^+^ cells (Figure 1A), suggesting that *Etnppl* is a highly specific marker of astrocytes in adult.

Previously, we showed that the expression level of *Etnppl* in the cervical cord tissue was significantly lower 3 days after pyramidotomy compared to sham in adults (Tsujioka and Yamashita, 2019). To histologically characterize *Etnppl*-expressing cells and their changes after pyramidotomy, we performed ISH using adult cervical cord tissues 3 days after pyramidotomy. A weak *Etnppl*-signal was detected in the gray matter of the adult spinal cord in both the pyramidotomy (Py) and sham groups (Figures 1B, C).

To analyze *Etnppl*-expressing cells more clearly, we performed an RNA scope assay, which is a highly sensitive ISH assay. The left side of the medullary pyramid, which can be visualized using the CST marker protein kinase C gamma (PKCγ) (Mori et al., 1990), was almost completely injured in these samples (Supplementary Figures 2A–F). Consistent with the qRT-PCR results (Figure 1A), *Etnppl* was almost undetectable in the neonates (Figures 1D, E and Supplementary Figures 2G, H). Consistent with the ISH results (Figures 1B, C), *Etnppl* was mainly expressed in the gray matter (Supplementary Figures 2I, J), suggesting the validity of both the ISH and RNAscope. *Etnppl*-expressing cells expressed the astrocyte marker *Aldh1l1* (Yang et al., 2011; Figures 1J–M) and did not express either the microglia/macrophage marker *chemokine (C-X3-C motif) receptor 1* (Harrison et al., 1998) (*Cx3cr1*; Figures 1N, O), or oligodendrocyte lineage marker *oligodendrocyte transcription factor 1* (Lu et al., 2000; Zhou et al., 2000) (*Olig1*; Figures 1P, Q), or pericyte marker *platelet derived growth factor receptor, beta polypeptide* (Winkler et al., 2010) (*Pdgfrb*; Figures 1R, S), in both Sham and Py groups; this suggests that *Etnppl* is selectively expressed in astrocytes under both conditions. To quantify whether the number of *Etnppl*^+^ cells changed after pyramidotomy, we set the region of interest (ROI) on the gray matter (Supplementary Figure 2K) and counted the number of *Etnppl*^+^ cells. The number of *Etnppl*^+^ cells significantly decreased after pyramidtomy in adult mice (Figures 1F–I, T), which was consistent with our previous study (Tsujioka and Yamashita, 2019). These results suggest that *Etnppl* is a novel marker for astrocytes in adult and reflects astrocytic conditions sensitive to pyramidotomy stimulation, which prompted us to examine *Etnppl* expression in other pathological conditions.

To examine changes in *Etnppl* expression under several pathological conditions, we analyzed previously published RNA-seq datasets. In a dataset produced by Chen et al. (2013), *Etnppl* was drastically downregulated after spinal cord injury (SCI) at the lesion site (Figure 2A). The downregulation was more prominent 2 days after injury (2D) compared to 7D, indicating that the *Etnppl*-response is acute. In a dataset produced by Androvic et al. (2020), *Etnppl* was significantly downregulated in the cortex 3 days after middle cerebral artery occlusion (MCAO) stroke (Figure 2B). In their dataset, *Etnppl*-expression was higher at 18 months (18M) than 3M, suggesting that *Etnppl*-expression continued to increase with age in mice even after 8 W.

We also examined previously published single-cell RNA-seq (scRNA-seq) datasets, which provide cellular-level information rather than tissue-level information. Because not all datasets provide metadata relating to cellular annotation, we created an automated annotation pipeline and performed pseudo bulk analysis in astrocytes. In the stroke model dataset produced by Shi et al. (2021), some of the clusters did not solely and selectively express their markers [*CD3 antigen, epsilon polypeptide* (*Cd3e*), T cell marker (Shi et al., 2021); *membrane-spanning 4-domains, subfamily A, member 7* (*Ms4a7*), macrophage marker (Milich et al., 2021); *natural killer cell group 7 sequence* (*Nkg7*), natural killer (NK) cell marker (Wu et al., 2021), *Fc receptor, IgE, high affinity I, alpha polypeptide* (*Fcer1a*), dendritic cell marker (Wu et al., 2021); *solute carrier family 4, sodium bicarbonate cotransporter, member 5* (*Slc4a5*), choroid plexus epithelial cell marker (Shi et al., 2021); *collagen, type I, alpha 1* (*Col1a1*), fibroblast marker (Milich et al., 2021); *Cx3cr1*, microglial marker; (Supplementary Figure 3B)]. However, the other cells, including astrocytes that are the focus of this study, selectively expressed their markers [*Lactotransferrin* (*Ltf*), neutrophil (granulocyte) marker (Milich et al., 2021); *RNA binding protein, fox-1 homolog 3* (*Rbfox3*), neuronal marker (Ikeda-Yorifuji et al., 2022); *platelet/endothelial cell adhesion molecule 1* (*Pecam1*), endothelial cell marker (Ikeda-Yorifuji et al., 2022); *Slc7a1* (Ikeda-Yorifuji et al., 2022) and *Aldh1l1*, astrocyte markers; *myelin oligodendrocyte glycoprotein* (*Mog*), oligodendrocyte marker; Ikeda-Yorifuji et al., 2022; Supplementary Figure 3B]. In a systemic inflammation model produced by intraperitoneal injection of lipopolysaccharide (LPS) by Hasel et al. (2021), all clusters (oligodendrocytes, microglia, neurons, endothelial cells, and astrocytes) selectively expressed these markers (Supplementary Figure 3C). These results indicate that our pipeline can be used for pseudobulk analysis of astrocyte clusters, although it may not be suitable for other analyses. *Etnppl* was selectively expressed in astrocyte clusters in these data and the SCI model dataset produced by Milich et al. (2021) (Supplementary Figures 3A–C), further supporting our results that *Etnppl* is selectively expressed in astrocytes. The expression level of *Etnppl* in astrocytes tended to decrease after SCI (Figure 2C), which was consistent with the results of the tissue-level dataset (Figure 2A), although it showed high variation and did not reach statistical significance. In the stroke model, *Etnppl* was significantly downregulated in astrocytes after ischemic stroke (Figure 2D), which was consistent with the results of tissue-level analysis (Figure 2B). Interestingly, the expression level of *Etnppl* tended to increase after hemorrhagic stroke, suggesting that *Etnppl* is not always downregulated under all pathological conditions but its response depends on the type of stimulation. Another example of *Etnppl* upregulation after stimulation was observed following intraperitoneal injection of LPS (Figure 2E). In this dataset, *Etnppl* expression was lower in male mice than in female mice. The above expression change was quite different from that of *Gfap*, a reactive astrocyte marker (Escartin et al., 2021). *Gfap* was upregulated not only after hemorrhagic stroke or intraperitoneal injection of LPS but also after SCI or MCAO (Supplementary Figures 3D–H), suggesting that *Etnppl* reflects astrocytic conditions that are different from those reflected by *Gfap* expression.

In our attempt to use an AAV vector for analysis of *Etnppl*, we found that the expression level of *Etnppl* significantly decreased 2 weeks after infection with an EGFP-expressing AAV serotype 8 control vector (Figure 2F). The same tendency was observed when serotype 5 was used. These results suggest that AAV infection itself affects *Etnppl*-expression. The expression level of *Etnppl* was nearly normal 2 weeks after pyramidotomy (Figure 2F), suggesting that the response of *Etnppl* is transient after pyramidotomy. In summary, *Etnppl* could be a novel marker of astrocytes in adult that is sensitive to several types of stimulation.

**FIGURE 3:**
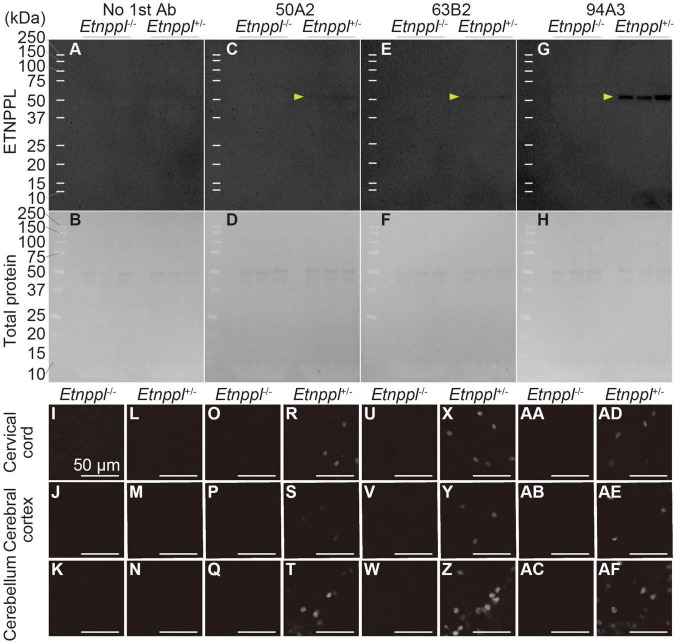
Specificity of anti-ETNPPL monoclonal antibodies. **(A–H)** Western blotting using anti-ETNPPL monoclonal antibodies. Black indicates the signal for ETNPPL **(A,C,E,G)** or the total protein **(B,D,F,H)**. Anti- ETNPPL monoclonal antibody clones 50A2 **(C,D)**, 63B2 **(E,F)**, or 94A3 **(G,H)** were used as primary antibody, or no primary antibody was applied **(A,B)**. Three samples were used for each group, and the lanes between each group or marker were empty lanes. Yellow arrowheads indicate the bands corresponding to the molecular weight of ETNPPL (55.5 kDa). **(I–AF)** Immunohistochemistry with anti-ETNPPL monoclonal antibodies. Enlarged images of the cervical cord **(I,L,O,R,U,X,AA,AD)**, cerebral cortex **(J,M,P,S,V,Y,AB,AE)**, and cerebellum **(K,N,Q,T,W,Z,AC,AF)** of *Etnppl*^–/–^ **(I–K,O–Q,U–W,AA–AC)** and *Etnppl*^+/–^ mice **(L–N,R–T,X–Z,AD–AF)** are shown. Clones 50A2 **(O–T)**, 63B2 **(U–Z)**, 94A3 **(AA–AF)** were applied, or no primary antibody was applied **(I–N)**. Scale bars: 50 μm.

To utilize *Etnppl* as a marker, the production of high-quality monoclonal antibody is very useful for the scientific community. We used His-tagged recombinant mouse ETNPPL as an antigen and produced rat monoclonal antibodies (Supplementary Figure 4B). We screened 95 clones and selected three clones that were suitable for IHC, western blotting (WB), or immunoprecipitation (IP; Supplementary Figures 4C–E). In our attempt to produce *Etnppl*-floxed mice, we fortuitously obtained *Etnppl*-knocked out (KO) mice (Supplementary Figures 4A, B) and used them to validate anti-ETNPPL monoclonal antibodies. A single band with a molecular weight (∼50 kDa) approximately corresponding to that of ETNPPL (55.5 kDa) was detected in *Etnppl*^+/–^ samples, and no band was detected in *Etnppl*^–/–^ samples in all three monoclonal antibody clones (Figures 3A–H), demonstrating that these antibodies are highly specific in WB. Consistent with our screening results, clone 94A3 showed the strongest signal on WB. In IHC, a clear signal was detected in *Etnppl*^+/–^ samples compared to *Etnppl*^–/–^ samples in all three monoclonal antibody clones (Figures 3I–AF and Supplementary Figure 5), demonstrating that these antibodies are highly specific for IHC. Consistent with our screening results, clone 63B2 exhibited the strongest IHC signal. Therefore, in the following histological analyses, we used clone 63B2. Application of clone 50A2 for IP was also confirmed with 10–20% efficiency (Supplementary Figure 4F).

**FIGURE 4:**
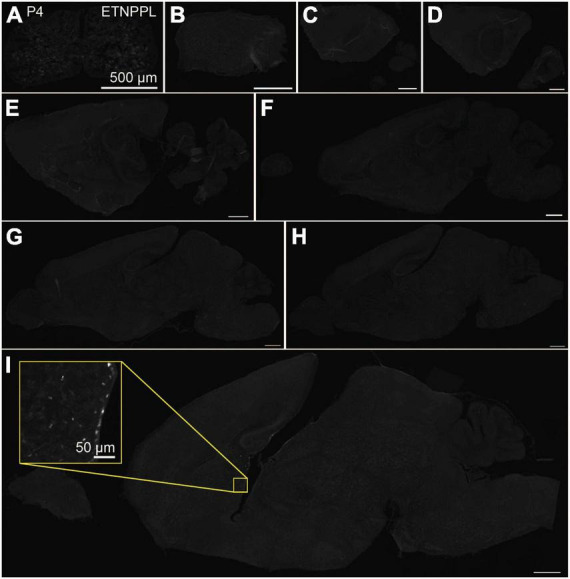
Localization of ETNPPL in the whole brain in P4. Coronal section of the cervical cord **(A)** or parasagittal section (approximately 320 μm interval) of the whole brain **(B–I)** of P4 female mice are shown. White indicates the ETNPPL signal revealed by IHC. Inset in a yellow box indicates an enlarged image. Scale bars: 50 μm for the inset or 500 μm for the other panels. The section images were appropriately rotated and cropped and the background was filled with black to minimize unnecessary space.

**FIGURE 5:**
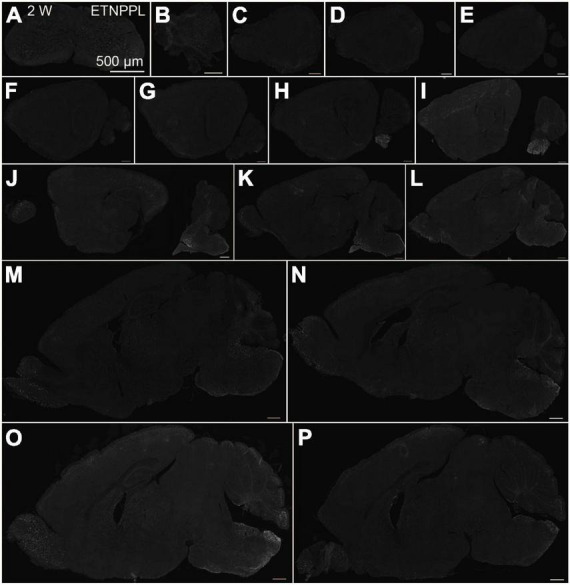
Localization of ETNPPL in the whole brain in 2-week old mice. Coronal section of the cervical cord **(A)**, or parasagittal section (approximately 250 μm interval) of the whole brain **(B–P)** of 2 W female mice are shown. White indicates the ETNPPL signal revealed by IHC. Scale bars: 500 μm. The section images were appropriately rotated and cropped and the background was filled with black to minimize unnecessary space.

**FIGURE 6:**
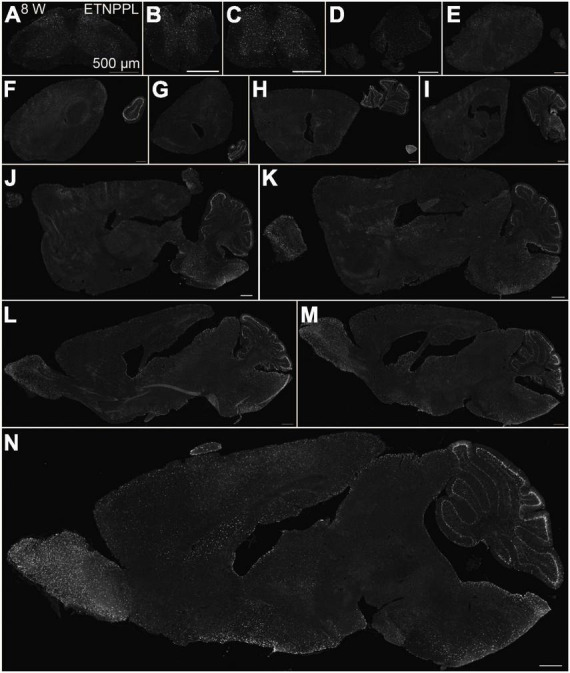
Localization of ETNPPL in the whole brain in 8-week old mice. Coronal sections of the cervical cord **(A)**, thoracic cord **(B)**, lumbar cord **(C)**, or parasagittal section (approximately 400 μm interval) of the whole brain **(D–N)** of 8 W female mice are shown. White indicates the ETNPPL signal revealed by IHC. Scale bars: 500 μm. The section images were appropriately rotated and cropped and the background was filled with black to minimize unnecessary space.

Using the anti-ETNPPL monoclonal antibody clone 63B2, we characterized ETNPPL-expressing cells in the whole brain of P4, 2 and 8 W female mice. Consistent with the above results for *Etnppl* mRNA, ETNPPL protein-expressing cells were almost undetectable at P4 (Figure 4 and Supplementary Figure 6). The exception was a weak but clear signal in the ventricular zone, and a very weak signal in the subventricular zone. At 2 W, a weak signal was observed in some areas of the brain (Figure 5 and Supplementary Figures 7, 8). Moderate signals were observed in the olfactory bulb and medulla; weak signals were observed in the ventricular zone, cerebellum, hypothalamus, thalamus, cerebral cortex, and spinal cord; and very weak signals were observed in the hippocampus and midbrain. At 8 W, the ETNPPL signal was detected throughout the brain, but unexpectedly, the distribution was not uniform (Figure 6 and Supplementary Figure 9). The signals were highest in the cerebellum, olfactory bulb, and hypothalamus and modest in the lateral septal nucleus, ventricular zone, pontine, medulla, midbrain, cerebral cortex, hippocampus, thalamus, and spinal cord. A very weak signal was detected in the white matter. In summary, ETNPPL expression increases as mice grow, and it is highly heterogeneous in the adult brain (summarized in Table 2).

**TABLE 2:** Summary of expression of ETNPPL in the whole brain.

	P4	2W	8W
+ +++			Cerebellum Olfactory bulb Hypothalamus
+++		Olfactory bulb Medulla	Lateral septal nucleus Ventricular zone Pontine Medulla Midbrain Cerebral cortex Hippocampus Thalamus Spinal cord
+ +	Ventricular zone	Ventricular zone Cerebellum Hypothalamus Thalamus Cerebral cortex Spinal cord	Other brain areas
+	Subventricular zone	Hippocampus Midbrain	White matter
−	Other brain areas, Spinal cord	Other brain areas	

**FIGURE 7:**
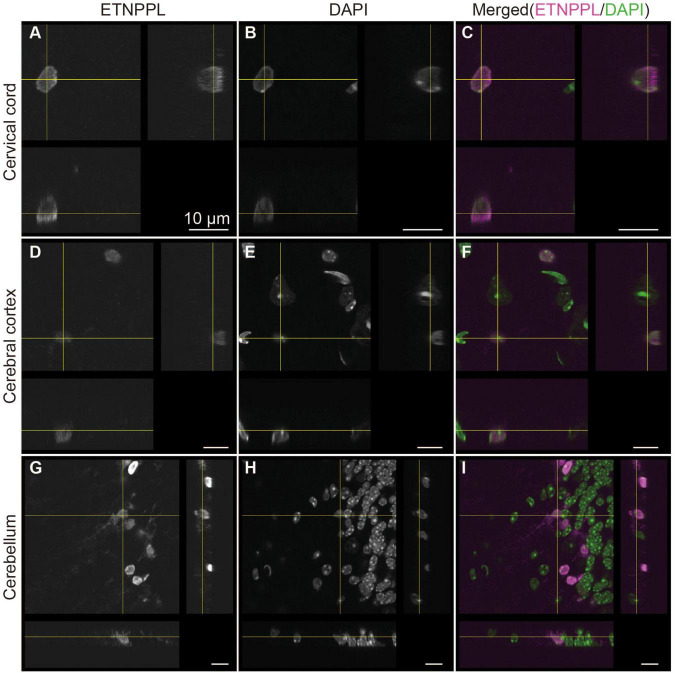
Subcellular localization of ETNPPL. Confocal orthogonal images of ETNPPL-expressing cells in the cervical cord **(A–C)**, cerebral cortex **(D–F)**, and cerebellum **(G–I)** in Figure 6 are shown. Signals for ETNPPL **(A,D,G)** or nuclear staining with DAPI **(B,E,H)** are shown. Merged images (magenta indicates ETNPPL and green indicates DAPI signal) are shown in **(C,F,I)**. Scale bars: 10 μm.

We also examined the subcellular localization of ETNPPL in some areas of the 8 W mice. A previous study reported that ETNPPL was localized in the cell nuclei or cytoplasm in the human cortex (Leventoux et al., 2020). In the mouse brain, we observed that ETNPPL was predominantly localized in the cell nuclei (Figure 7), which is suitable for cell counting. Localization in the cytoplasm was very rare and weak (an example is shown with yellow crosses in Figures 7D–F). An exception was the cerebellum (Figures 7G–I). A relatively clear signal was detected in fibers that looked like Bergman fibers, although even in that case, the signal was weaker than the signal in the nucleus.

**FIGURE 8:**
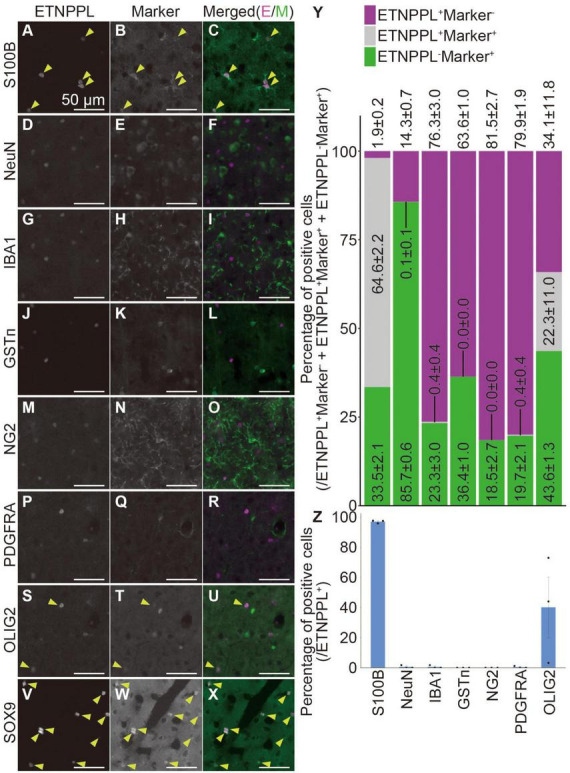
Cellular specificity of ETNPPL-expressing cells in the cerebral cortex of adult mice. **(A–X)** Representative images of multiple staining of ETNPPL and cell markers in the cerebral cortex of 8 W female mice. Signals for ETNPPL **(A,D,G,J,M,P,S,V)**, S100B **(B)**, NeuN **(E)**, IBA1 **(H)**, GSTπ **(K)**, NG2 **(N)**, PDGFRA **(Q)**, OLIG2 **(T)**, or SOX9 **(W)** and their merged images **(C,F,I,L,O,R,U,X)** are shown. In the merged images, magenta indicates the signal for ETNPPL, and green indicates the signal for cell markers. Yellow arrowheads indicate cells double positive for ETNPPL and cell markers. Some experiments were not performed on the same day, and the brightness of the ETNPPL channels was adjusted differently. Scale bars: 50 μm. The same ETNPPL channel images were used in **(D,G,F,I,P,R,S,U)**. **(Y,Z)** Quantification of the percentage of **(Y)** cells expressing ETNPPL and the cell markers or **(Z)** ETNPPL^+^ cells labeled with a certain cell-type specific marker. Magenta indicates ETNPPL single positive cells, green indicates cell marker single positive cells, and gray indicates cells that are double positive for ETNPPL and cell markers. Mean ± S.E.M. *n* = 3 individuals, each of which is the average of three different sections.

**FIGURE 9:**
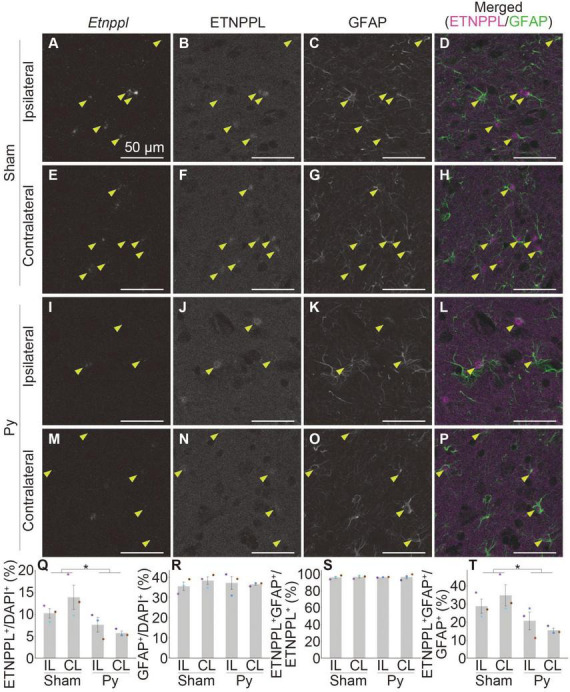
Astrocytic response after pyramidotomy revealed by IHC using anti ETNPPL monoclonal antibody. **(A–P)** Representative confocal images of the ipsilateral **(A–D,I–L)** or contralateral side **(E–H,M–P)** of the gray matter of the cervical cord of 8 W female mice 3 days after sham surgery **(A–H)** or pyramidotomy **(I–P)**. Signals for *Etnppl* mRNA (RNAscope; **A,E,I,M**), ETNPPL protein **(B,F,J,N)**, and the retrieved GFAP protein **(C,G,K,O)** are shown. In merged images **(D,H,L,P)**, magenta indicates the signal for ETNPPL protein and green indicates the signal for GFAP. Yellow arrowheads indicate double-positive cells for ETNPPL protein and GFAP. **(Q–T)** Quantification of changes in ETNPPL-expressing cells after pyramidotomy. Vertical axes represent the percentage of ETNPPL^+^ cells to total cells **(Q)**, GFAP^+^ astrocytes to total cells **(R)**, ETNPPL^+^GFAP^+^ cells to total ETNPPL^+^ cells **(S)**, or ETNPPL^+^GFAP^+^ cells to total GFAP^+^ astrocytes **(T)**. Mean ± S. E. M. *n* = 3 individuals, each of which is the average of four different sections. *P < 0.05, Sham vs. Py, two-way ANOVA. The same color indicates the same individual. IL, ipsilateral; CL, contralateral.

To examine whether the monoclonal antibody we produced is useful for specifically labeling astrocytes, we quantified the number of cells expressing ETNPPL or other cell markers. We focused on the cerebral cortex (Supplementary Figure 2L) and cervical cords of 8 W mice as representatives of the two different regions of the CNS, the brain, and the spinal cord. In the cerebral cortex, ETNPPL was almost not expressed in cells expressing the neuronal marker neuronal nuclei (Mullen et al., 1992) (NeuN; Figures 8D–F, Y, Z), microglial marker ionized calcium-binding adapter molecule 1 (Ito et al., 1998) (IBA1; Figures 8G–I, Y, Z), the oligodendrocyte marker glutathione S-transferase pi (Tansey and Cammer, 1991) (GSTπ; Figures 8J–L, Y, Z), oligodendrocyte precursor cell (OPC) and pericyte marker neuron-glial antigen 2 (Stallcup and Beasley, 1987) (NG2; Figures 8M–O, Y, Z), or OPC marker PDGFR alpha (Hall et al., 1996) (PDGFRA; Figures 8P–R, Y, Z), suggesting that ETNPPL is not expressed in neurons, microglia, oligodendrocytes, OPC, or pericytes. Almost all ETNPPL^+^ cells expressed astrocytic marker S100B (Boyes et al., 1986; Figures 8A–C, Y, Z). Although S100B is thought to be expressed also in some neurons in rats (Rickmann and Wolff, 1995), considering the results above, these results suggest that ETNPPL is selectively expressed in astrocytes. ETNPPL signal also colocalized with a nuclear-localized astrocytic marker SRY (sex determining region Y)-box 9 (SOX9) (Sun et al., 2017) signal (Figures 8V–X). A recent study reported that contrary to the traditional view that OLIG2 is an oligodendrocyte lineage marker (Lu et al., 2000; Zhou et al., 2000), it is also expressed in approximately 10–30 or 90–100% of astrocytes in the cortex or the spinal cord, respectively (Wang et al., 2021). We observed that approximately 40% of ETNPPL^+^ cells were positive for OLIG2 (Figures 8S–U, Y, Z), suggesting that part of ETNPPL^+^ cells were OLIG2^+^ astrocytes. Similar results were obtained for the cervical cord (Supplementary Figure 10). ETNPPL was almost not expressed in NeuN^+^ cells (Supplementary Figures 10A–C, S, U), IBA1^+^ cells (Supplementary Figures 10D–F, S, U), GSTπ^+^ cells (Supplementary Figures 10G–I, S, U), NG2^+^ cells (Supplementary Figures 10J–L, S, U), or PDGFRA^+^ cells (Supplementary Figures 10M–O, S, U). The signal for the astrocyte marker GFAP (Eng et al., 1971) is usually not detected in the gray matter of the spinal cord, but we fortuitously found that the GFAP signal is enhanced by target retrieval treatment of the RNAscope assay, and the signal can be detected in gray matter. Almost all ETNPPL^+^ cells were positive for GFAP (Figure 9 and Supplementary Figures 10T, V). Almost all ETNPPL^+^ cells were OLIG2^+^ (Supplementary Figures 10P–S, U). In the olfactory bulb, almost all ETNPPL^+^ cells were GFAP^+^ (Supplementary Figures 11A–D). In the cerebellum, ETNPPL^+^ in the Purkinje cell layer were SOX9^+^ (Supplementary Figures 11E–G), suggesting that the cells are Bergmann glia. The above results suggest that ETNPPL can be used as a marker of astrocytes in adult both in the cerebral cortex and spinal cord, and the monoclonal antibody we produced is useful for labeling ETNPPL^+^ astrocytes.

We then examined whether the monoclonal antibody is useful for detecting changes in astrocytes in pathological conditions by analyzing pyramidotomy samples used in Figures 1D–T. In addition to the above purpose, to examine whether the antibody can be used in combination with IHC and RNAscope assays and to further validate the specificity of the *Etnppl* RNAscope probe and anti-ETNPPL monoclonal antibody, we performed a combination of IHC for ETNPPL and RNAscope for *Etnppl*. We also added anti-GFAP to label total astrocytes in this assay. Although the ETNPPL signal in this assay was weaker than that in IHC alone, the ETNPPL signal could still be detected in this assay (Figures 9B, F, J, N), indicating that the monoclonal antibody is also useful in combination with RNAscope. Many *Etnppl* RNAscope signals were detected in ETNPPL expressing cells (Figures 9A, B, E, F, I, J, M, N), further validating the specificity of both the *Etnppl* RNAscope probe and anti-ETNPPL monoclonal antibody. The number of ETNPPL-expressing cells significantly decreased after pyramidotomy (Figures 9B, F, J, N, Q), which was consistent with the reduction in the number of *Etnppl* mRNA-expressing cells (Figure 1T). The number of GFAP^+^ cells did not change significantly (Figures 9C, G, K, O, R). Percentage of ETNPPL^+^GFAP^+^ cells to ETNPPL^+^ cells was almost 100% in both Sham and Py groups and did not change significantly (Figures 9D, H, L, P, S). The percentage of ETNPPL^+^GFAP^+^ cells among the GFAP^+^ total astrocytes significantly decreased after pyramidotomy (Figure 9T). Taken together, these results imply that the reduction in ETNPPL^+^ cells might be explained by the conversion of ETNPPL^+^ astrocytes into ETNPPL^–^ astrocytes. The above results also suggest that the antibody we produced is useful for detecting astrocytic changes under pathological conditions.

**FIGURE 10:**
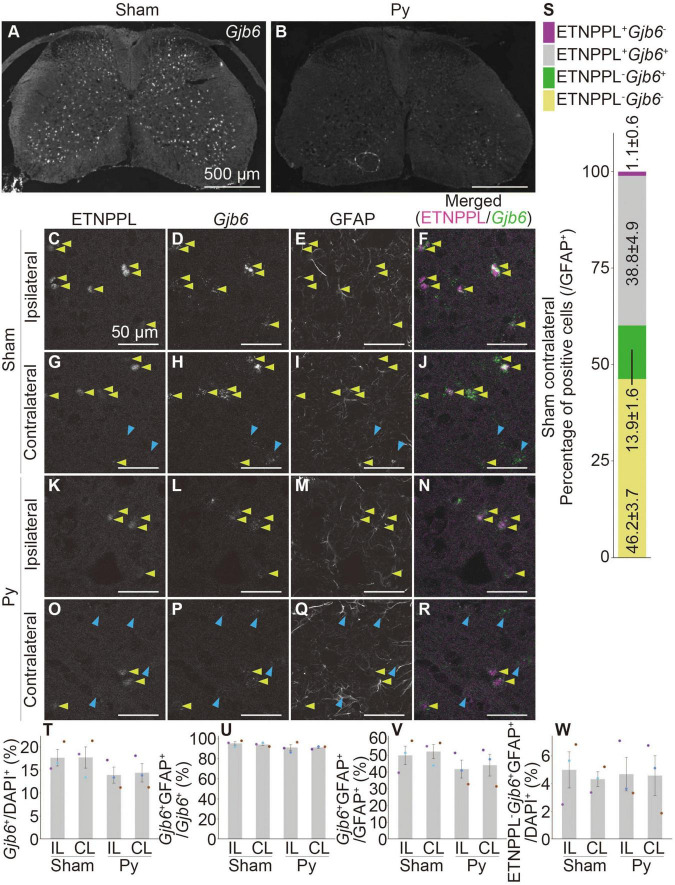
Co-localization of ETNPPL and *Gjb6*. **(A,B)** Representative images of the cervical cord of 8 W female mice 3 days after sham surgery **(A)** or pyramidotomy **(B)** stained for *Gjb6* (RNAscope). Scale bars: 500 μm. **(C–R)** Magnified confocal images of the gray matter of the ipsilateral **(C–F,K–N)** or contralateral side **(G–J,O–R)** of the sham **(C–J)** or pyramidotomy group **(K–R)** are shown. Signals for ETNPPL **(C,G,K,O)**, *Gjb6* **(D,H,L,P)**, or retrieved GFAP **(E,I,M,Q)** are shown. In merged images **(F,J,N,R)**, magenta indicates ETNPPL and green indicates *Gjb6*. Yellow arrowheads indicate the ETNPPL-*Gjb6*-GFAP triple-positive cells. Blue arrowheads indicate the ETNPPL^–^*Gjb6*^+^GFAP^+^ cells. Scale bars: 50 μm. **(S)** Percentage of each cell population among total GFAP^+^ astrocytes on the contralateral side of the sham group. Magenta indicates ETNPPL^+^*Gjb6*^–^ cells, gray indicates ETNPPL^+^*Gjb6*^+^ cells, green indicates ETNPPL^–^*Gjb6*^+^ cells, and yellow indicates ETNPPL^–^*Gjb6*^–^ cells. **(T–W)** Quantification of changes in *Gjb6*-expressing cells after pyramidotomy. The vertical axes represent the percentage of *Gjb6*^+^ cells to total cells **(T)**, *Gjb6*^+^GFAP^+^ cells to total *Gjb6*^+^ cells **(U)**, *Gjb6*^+^GFAP^+^ cells to total GFAP^+^ astrocytes **(V)**, or ETNPPL^–^*Gjb6*^+^GFAP^+^ cells to total cells **(W)**. Mean ± S. E. M. *n* = 3 individuals, each of which is the average of four different sections. The same color indicates the same individual. IL, ipsilateral; CL, contralateral.

The highly heterogeneous distribution of ETNPPL shown in Figure 6 suggests that ETNPPL is expressed in a subpopulation of astrocytes. In the Source Data for Figure 10F of the previous study by Shi et al. (2021), *Etnppl* was listed as a marker of cluster 2. In this cluster, genes involved in synaptic plasticity or gap junctions, such as *angiotensinogen (serpin peptidase inhibitor, clade A, member 8)* (*Agt*) or *Gjb6*, are highly expressed. In Figure 1C of the previous study by Hasel et al. (2021), *Etnppl* is a marker of cluster 7, in which genes involved in synapse modulation or AMPA receptors such as *secreted acidic cysteine rich glycoprotein* or *glutamate receptor, ionotropic, AMPA1 (alpha 1)* are highly expressed. Strikingly, spatial transcriptomic analysis of the coronal section in Figure 1D of the study indicated that cluster 7 was enriched in the hypothalamus, which is consistent with our result that the expression of ETNPPL is highest in the hypothalamus (Figure 6, Table 2, and Supplementary Figure 5). The results of previous studies prompted us to examine whether the genes described are truly expressed in ETNPPL^+^ cells. We focused on *Agt* [which is known to promote axonal sprouting after SCI (Namsolleck et al., 2013)], *Gjb6* [which is known to be responsible for closure of the critical period of the visual cortex (Ribot et al., 2021)], and *inter-alpha trypsin inhibitor, heavy chain 3* (*Itih3*) [which might be involved in psychiatric diseases (Cardno and Owen, 2014; Goulding et al., 2019) and is listed as a marker of cluster 7 in the study by Hasel et al. (2021)]. By searching for them in the previously published dataset, we found that all of them were specifically expressed in astrocytes (Supplementary Figures 3A–C). *Itih3* was significantly downregulated after SCI, and *Gjb6* and *Agt* showed the same tendency (Supplementary Figures 12A, D, G). All of them were significantly upregulated after intraperitoneal injection of LPS (Supplementary Figures 12C, F, I). *Agt* and *Itih3* was significantly upregulated after hemorrhagic stroke and *Gjb6* showed the same tendency (Supplementary Figures 12B, E, H). These were consistent with the change in *Etnppl* expression (Figures 2C–E). *Gjb6* was significantly downregulated after ischemic stroke (Supplementary Figure 12B), which was consistent with the change in *Etnppl* expression (Figure 2D). In contrast, *Agt* and *Itih3* were significantly upregulated after ischemic stroke (Supplementary Figures 12E, H), implying that these genes might not perfectly represent the ETNPPL-expressing subpopulation.

We performed a combination of IHC for ETNPPL and RNAscope for these genes. *Gjb6* and *Itih3* were mainly expressed in the gray matter (Figures 10A, B and Supplementary Figures 13G, H), which was consistent with the localization of ETNPPL (Figure 6). In contrast, the signal for *Agt* was stronger in the white matter than that in the gray matter (Supplementary Figures 13A, B), suggesting that the expression of *Agt* is not limited to ETNPPL^+^ cells. Many cells expressing these genes also expressed ETNPPL in the gray matter (Figures 10C–R and Supplementary Figures 13C–F, I–L). We focused on *Gjb6* and counted the number of these cells. Almost all ETNPPL^+^ astrocytes were *Gjb6*^+^, and approximately three-quarters of *the Gjb6*^+^ astrocytes were ETNPPL^+^ (Figure 10S), indicating that *Gjb6*^+^ astrocytes included ETNPPL^+^ astrocytes. The number of *Gjb6*^+^ cells tended to decrease after pyramidotomy, although the difference was not statistically significant (Figure 10T). The percentage of *Gjb6*^+^GFAP^+^ to total *Gjb6*^+^ cells was approximately 90%, and it did not change significantly after pyramidotomy (Figure 10U), suggesting that most *Gjb6*^+^ cells were astrocytes. The percentage of *Gjb6*^+^GFAP^+^ cells to total GFAP^+^ cells tended to decrease after pyramidotomy, although the difference was not significant (Figure 10V). Consistent with it, the number of dots of *Gjb6* signal tended to be lower in pyramidotomy group, but the difference did not reach statistical significance (Supplementary Figure 14A) due to high variation. The number of ETNPPL^–^*Gjb6*^+^GFAP^+^ cells did not change significantly after pyramidotomy (Figure 10W and Supplementary Figures 14B–R). Although no statistically significant difference does not mean no difference, these results might imply the possibility that the number of ETNPPL^–^*Gjb6*^+^ astrocytes does not change after pyramidotomy, and it obscures the change of that of total *Gjb6*^+^ cells. Therefore, *Gjb6* might also be a marker for detecting astrocytic responses after pyramidotomy, but further analyses are needed to clarify it.

## 4. Discussion

In this study, we showed that *Etnppl* is selectively expressed in astrocytes in adult, and its expression levels change in different directions depending on the type of stimulation. We created high-quality monoclonal antibodies against ETNPPL and showed that ETNPPL is heterogeneously expressed in the brain, with cell nuclear-dominant localization. We also showed that the antibody was useful for labeling a subset of astrocytes in adult and detecting astrocytic changes after pyramidotomy. Finally, we showed that ETNPPL was expressed in a subset of *Gjb6* expressing cells. This is the first study to examine the potential of ETNPPL as an astrocytic marker, develop high-quality monoclonal antibodies against ETNPPL, and show their potential usefulness in astrocytic research.

*Etnppl* was previously called *alanine-glyoxylate aminotransferase 2-Like 1* (*Agxt2l1*). At present, there are very few studies relating to *Etnppl* or *Agxt2l1* (less than 20 hits each in PubMed in September 2022). However, some studies have reported interesting relationships between *Etnppl* and CNS disease. The expression level of *ETNPPL* negatively correlates with the progression of some gliomas (Leventoux et al., 2020), suggesting the potential of *Etnppl* as a glioma marker. Overexpression of ETNPPL in a glioma reduces its proliferation, suggesting that ETNPPL regulates glioma proliferation (Leventoux et al., 2020). A transcriptomic study reported *ETNPPL* as one of the top genes upregulated in schizophrenia or bipolar patients (Shao and Vawter, 2008), and a re-analysis of 751 transcriptomic samples of stress-induced depression models identified *Etnppl* as the second highest group that showed constant expression changes in different samples (overlap among 18 BioProjects is 5) (Flati et al., 2020), suggesting that *Etnppl* is involved in psychiatric diseases. Taken together with the results of the present study that showed the expression level of *Etnppl* changes in SCI, stroke, or systemic inflammation (Figure 2), *Etnppl*-expressing cells might be involved in diverse CNS diseases, including cancer, psychiatric diseases, injury, stroke, or inflammation, and might be a worse investigation in future analyses. This highlights the importance of producing high-quality monoclonal antibodies (Figure 3), which will be useful in future studies.

The opposite regulation of *Etnppl* between ischemic stroke and LPS injection models (Figures 2D, E) is consistent with a previous study showing that the gene expression profile of astrocytes changes differently between MCAO and LPS injection models (Zamanian et al., 2012). Originally, the authors thought that LPS-induced astrocytes were detrimental based on their gene expression profile, and according to this assumption, *Etnppl*-expressing cells might be a detrimental population. However, a recent consensus statement in the astrocytic research field strongly argues against the overly simplified view that one population is detrimental, and the other population is beneficial (Escartin et al., 2021). Thus, the functions of *Etnppl*-expressing cells must be analyzed under each pathological condition. The different regulations between ischemic stroke and hemorrhagic stroke are also interesting (Figure 2D). Different astrocytic responses between stroke subtypes were also observed in a previous study, in which genes related to phagocytosis were downregulated in hemorrhagic stroke compared to ischemic stroke (Shi et al., 2021), suggesting that distinction of stroke subtypes is necessary to understand astrocytic response. Taken together, many studies will be conducted to reveal the context-dependent function of *Etnppl*-expressing cells in various pathological conditions, and we believe that the monoclonal antibodies we produced will be useful for these studies.

*Etnppl* was downregulated after control AAV infection (Figure 2F). Because the AAV vector expresses only EGFP, the downregulation is probably induced by AAV infection itself. Although the immunogenicity of AAV is thought to be low, some immune responses are known to be induced by AAV infection (Rabinowitz et al., 2019). Considering that the expression of *Etnppl* changes after LPS injection, the downregulation of *Etnppl* after AAV infection might be related to an immune response induced by AAV. Thus, control should be carefully chosen when AAV is used to analyze something related to *Etnppl*.

In this study, we produced high-quality monoclonal antibodies (Figure 3 and Supplementary Figures 4, 5). A polyclonal antibody for ETNPPL used in a previous study did not detect signals for ETNPPL in the hippocampus of mice (Wang et al., 2022), but we detected a relatively weak but clear signal in the hippocampus (Figure 6), suggesting a higher sensitivity of our antibodies compared to a commercially available anti- ETNPPL antibody. Since the antibodies we produced were monoclonal antibodies, general merits of monoclonal antibodies, which comprise the same immunoglobulin, are also expected for our antibodies, such as lower background and higher reproducibility than polyclonal antibodies. Moreover, as we validated the specificity of the antibodies in this study, these antibodies will be useful and reliable resources for the scientific community.

Although the expression of ETNPPL was very weak in P4 in general, an exception was observed in the ventricular and subventricular zones (Figure 4 and Table 2). Since these zones are well-known neurogenic/gliogenic niches (Luskin, 1998; Zheng et al., 2022), ETNPPL-expressing cells in these zones might not be astrocytes, although we confirmed the astrocyte-selective expression of ETNPPL in the cerebral cortex (Figure 8) or spinal cord (Supplementary Figure 10) in adults. A recent study found that ETNPPL^+^ cells express the neural stem cell (NSC) marker (SOX2) or a neural progenitor cell marker (neurogenic differentiation 1), as well as a proliferation marker (proliferating cell nuclear antigen) in the adult hippocampus in primates but not in rodents (Wang et al., 2022). This suggests possible expression of ETNPPL in hippocampal NSC only in primates. Therefore, ETNPPL may also be expressed in a subset of NSCs in mice, as GFAP is also expressed in adult NSC (Götz et al., 2015). Further studies are needed to characterize ETNPPL-expressing cells in the embryonic or neonatal brain and the inter-species differences. The discrepancy that on one hand ETNPPL^+^ cells express mature astrocytic genes, and that on the other hand, they express neurogenic genes might reflect spatial specification of them. Since neurogenic/gliogenic niches are restricted to specific brain areas, molecular cues in the niches might change the phenotype of ETNPPL^+^ cells to neurogenic whereas those in the other brain areas might direct the phenotype of ETNPPL^+^ cells to mature astrocyte. Further studies are required to comprehensively understand both aspects of ETNPPL^+^ cells.

In the present study, we found that ETNPPL was most prominently expressed in the olfactory bulb, hypothalamus, and cerebellum of adults (Figure 6 and Table 2). Among them, the hypothalamus is known as a center for the regulation of food intake, and the olfactory bulb, which is necessary for food-seeking behavior as a center of the olfaction system, is suggested to be associated with the hypothalamus and appetite (Fine and Riera, 2019). The cerebellum is also connected to the hypothalamus, and is involved in the regulation of food intake (Zhu and Wang, 2008). Interestingly, a previous study showed that *Etnppl* was upregulated by fasting (White et al., 2021). Considering these studies, ETNPPL^+^ cells in the olfactory bulb, hypothalamus, and cerebellum might be involved in the connection between the nutritional needs of the body and the regulation of food intake under physiological conditions.

We found that ETNPPL was predominantly expressed in the cell nuclei (Figure 7). This was unexpected from the biochemical properties of ETNPPL. ETNPPL catalyzes the degradation of ethanolamine phosphate (Schiroli et al., 2013), and the phospholipid profile in the brain changes if *Etnppl* is KO (White et al., 2021). The above function does not require the nuclear localization of ETNPPL. ETNPPL might modulate the nuclear envelope, or products of its enzymatic reaction (acetaldehyde, phosphate, or ammonium) might modulate the nuclear function. Another possibility is that ETNPPL itself has a function other than the enzyme, called the moonlighting effect (Boukouris et al., 2016), and modulates nuclear function. Since the monoclonal antibody we produced can also be used for IP (Supplementary Figure 4F), it might be useful to analyze the possible interactions between ETNPPL and other molecules.

In this study, we found that ETNPPL is selectively expressed in astrocytes in adult (Figures 4–6), and the ratio of ETNPPL^+^ astrocytes to total astrocytes decrease after pyramidotomy (Figure 9). This raises the possibility that mature astrocytes are converted to a relatively immature state after pyramidotomy. We also found that ETNPPL^+^ astrocytes are a subset of *Gjb6*^+^ astrocytes (Figure 10). Interestingly, *Gjb6* (Ribot et al., 2021) is expressed selectively in mature astrocytes, and it is thought to downregulate matrix metalloproteinase 9 through interaction with molecules relating to Ras homolog A signaling and stabilize perineuronal nets of parvalbumin-positive cells, leading to a reduction in neuronal plasticity and eventual closure of the critical period (Ribot et al., 2021). Thus, the ETNPPL^+^ subpopulation of *Gjb6*^+^ astrocytes may also possess the ability to inhibit neural circuit organization. Taken together, it is possible that a subset of the ETNPPL^+^ subpopulation of *Gjb6*^+^ mature astrocytes is converted to a relatively immature state, which permits neural circuit reorganization and promotes axonal sprouting after pyramidotomy. Contrary to the assumption that ETNPPL^+^ cells inhibit neural circuit repair, the axonal-sprouting-promoting factor *Agt* was also expressed in ETNPPL^+^ cells (Supplementary Figures 13C–F). *Agt* encodes a precursor of angiotensin II, a component of the renin-angiotensin system. As a tissue renin-angiotensin system, stimulation of the AT2-receptor enhances axonal sprouting after SCI (Namsolleck et al., 2013). ETNPPL^+^ cells probably play multiple roles, which should be revealed by functional analyses in each context in future studies.

The expression of *Itih3* in ETNPPL^+^ cells (Supplementary Figures 13I–L) is also interesting. The genomic region, including the *Itih3* locus, is associated with schizophrenia and bipolar disorder (Cardno and Owen, 2014), and *Itih3* is listed in the second highest group, which shows constant expression changes in different stress-induced depression models with *Etnppl* (Flati et al., 2020), suggesting the involvement of *Itih3* in psychiatric diseases. A previous study found that deletion of the *alpha 1 microglobulin/bikunin precursor*, which is necessary for the functional complex of ITIH1 or ITIH3, increases anxiety behavior (Goulding et al., 2019), suggesting the involvement of *Itih3* in mood disorders. Interestingly, ITIH is thought to stabilize hyaluronan (Huang et al., 1993), which is a component of the extracellular matrix and perineuronal nets (Chelyshev et al., 2022). Considering that *Gjb6* modulates neuronal plasticity by regulating the stability of perineuronal nets (Ribot et al., 2021), *Itih3* may also modulate neuronal plasticity. Since neuronal plasticity is thought to be altered in mood disorders (Carlson et al., 2006) and antidepressants affect astrocytes (Di Benedetto et al., 2013), it is possible that *Itih3* in ETNPPL^+^ astrocytes might be involved in mood disorders or other psychiatric diseases through the regulation of neuronal plasticity.

The present study has a limitation. Intensity was not considered in the quantification of the histological samples. High variation in fluorescence intensity, especially in the RNAscope assay, hindered the accurate quantification of fluorescence intensity. Therefore, we only quantified the number of all positive cells manually, ranging from weakly positive to strongly positive. Since the intensity of ETNPPL is not uniform, categorizing ETNPPL-positive cells depending on the intensity of ETNPPL would be more informative. For this purpose, more accurate methods to detect differences in intensity, such as FACS, may be more suitable.

## Data availability statement

The datasets presented in this study can be found in online repositories. The names of the repository/repositories and accession number(s) can be found in the article/Supplementary material.

## Ethics statement

All animal experiments were approved by the Animal Experiment Committee of Osaka University (permission numbers 29-058-078 and 01-013-055).

## Author contributions

HT performed all experiments and wrote the manuscript. TY supervised the research, interpreted the results, and revised the manuscript. Both authors have read and approved the manuscript.
