# Supplementary material for: Utilization of ethanolamine phosphate phospholyase as a unique astrocytic marker
Source: Front Cell Neurosci. 2023 Jan 30;17:1097512. doi: 10.3389/fncel.2023.1097512 (PMC9922850; doi:10.3389/fncel.2023.1097512)
Supplement: Supplementary file 1 [file Data_Sheet_1.pdf]

Supplementary Material

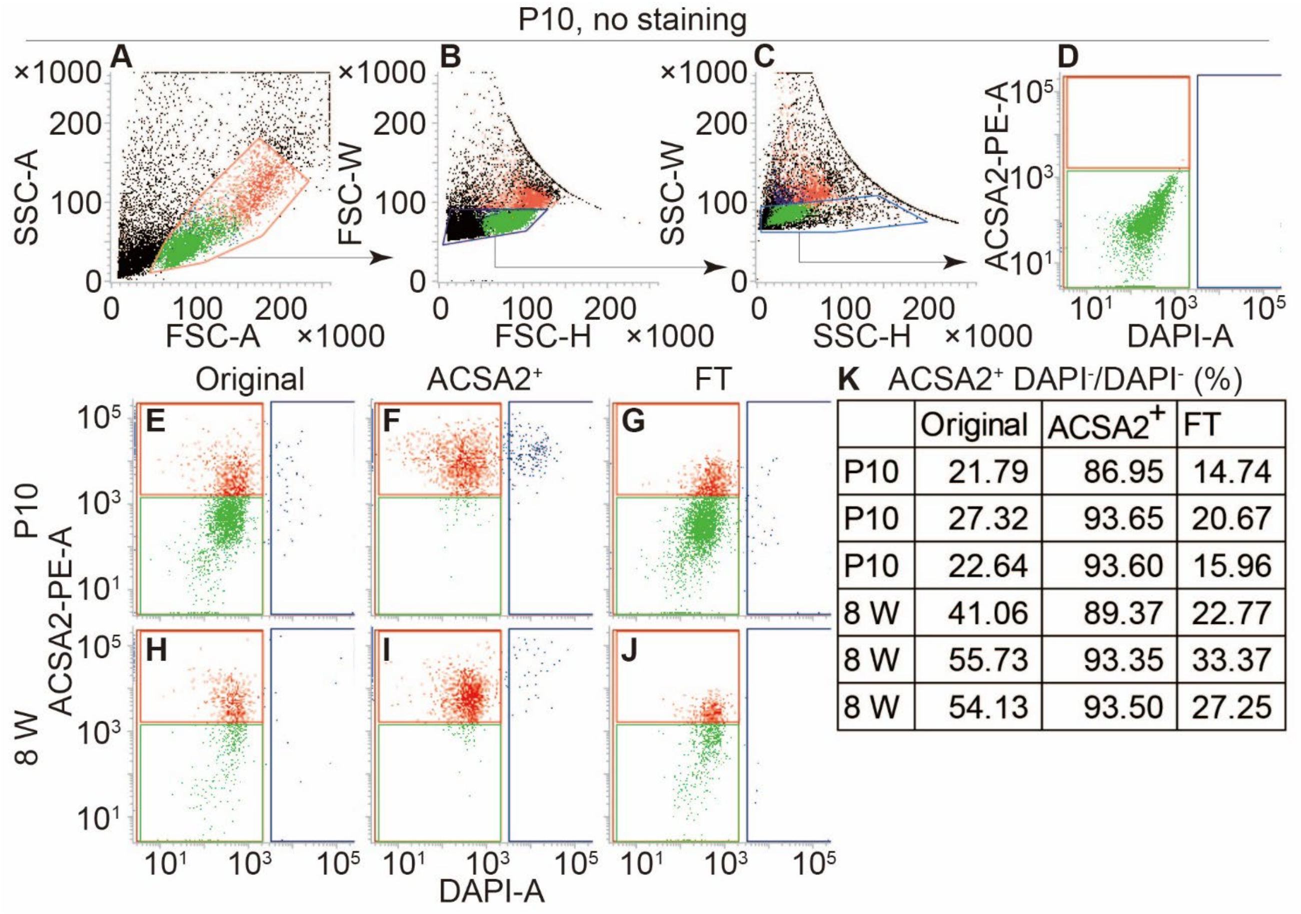

	Original	ACSA2$^+$	FT
P10	21.79	86.95	14.74
P10	27.32	93.65	20.67
P10	22.64	93.60	15.96
8 W	41.06	89.37	22.77
8 W	55.73	93.35	33.37
8 W	54.13	93.50	27.25

Supplementary Figure 1 Purity of MACS sorted astrocyte.

A-D Representative FACS gating strategy. The result using the P10 no-staining sample is shown. SSC, side scatter; FCS, forward scatter; A, area; W, width; H, height; PE, phycoerythrin. **E-J** Representative dot plot of FACS results. The original cell suspension (E, H), ACSA2$^+$ fraction (F, I), or flow-through (FT; G, J) harvested from the spinal cord of P10 (E-G) or 8 W (H-J) mice are shown. **K** Percentage of ACSA2$^+$DAPI$^-$ live astrocytes to total DAPI$^-$ live cells.

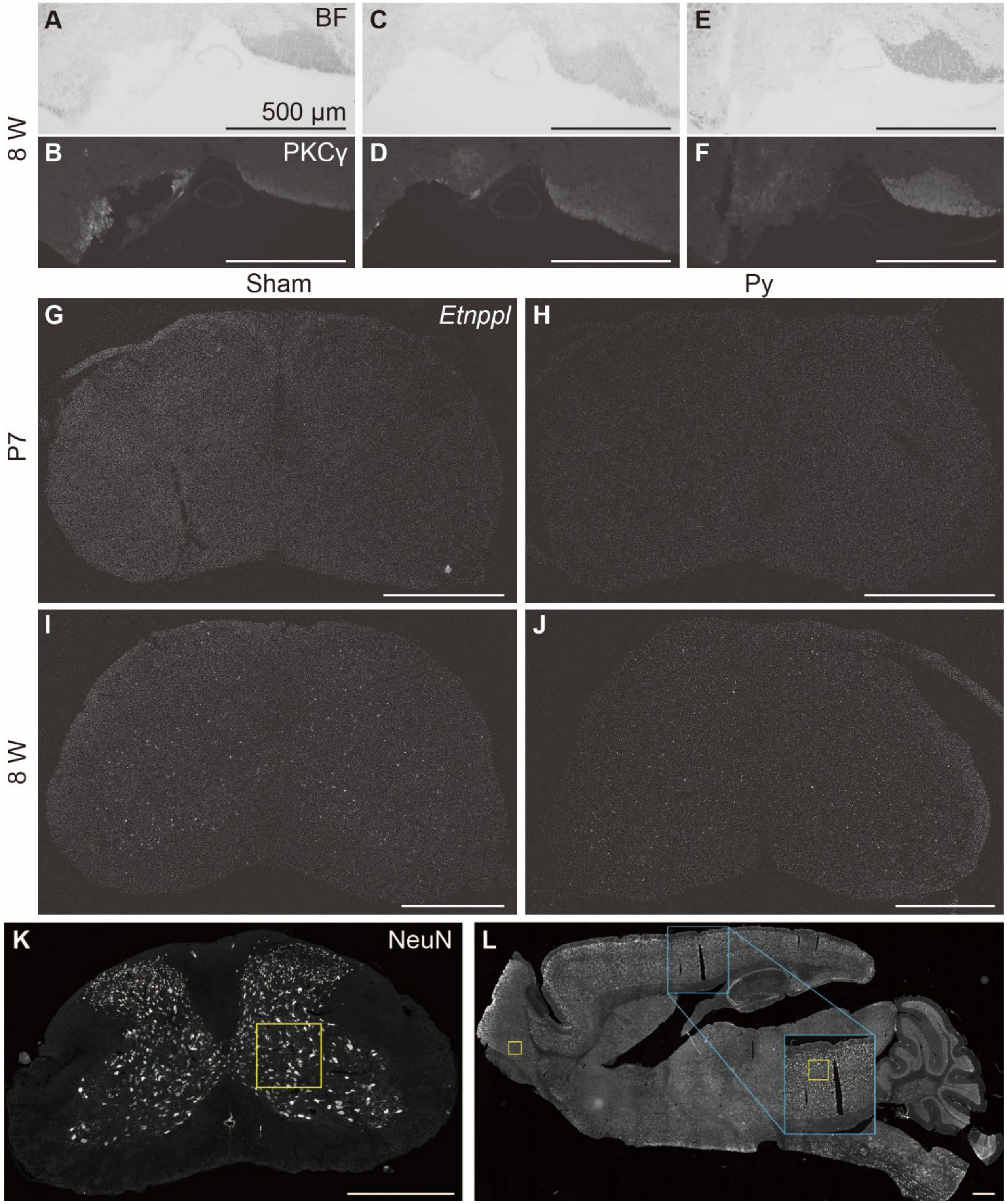

Supplementary Figure 2 Samples used for histological analyses.

A-F Validation of the pyramidotomy model used in RNAscope assays. Bright field (BF) images (A, C, E) or signals for corticospinal tract marker PKCγ (B, D, F) at the lesion (medullary pyramids) of three Py group individuals used for quantification are shown. Dorsal is top, left is to the left. **G-J**

Confocal images of the RNAscope signal for *Etnppl* in the cervical cord. Coronal sections of female mice subjected to sham surgery (G, I) or pyramidotomy (H, J) at P7 (G, H) or 8 W (I, J) 3 days before fixation are shown. **K, L** Representative region of interest (ROI) used for quantification in the cervical cord (K) or cerebral cortex or olfactory bulb (L) in this study. Yellow boxes indicate ROI used for quantification. NeuN signals are used to clarify the anatomical location. Scale bars: 500 µm.

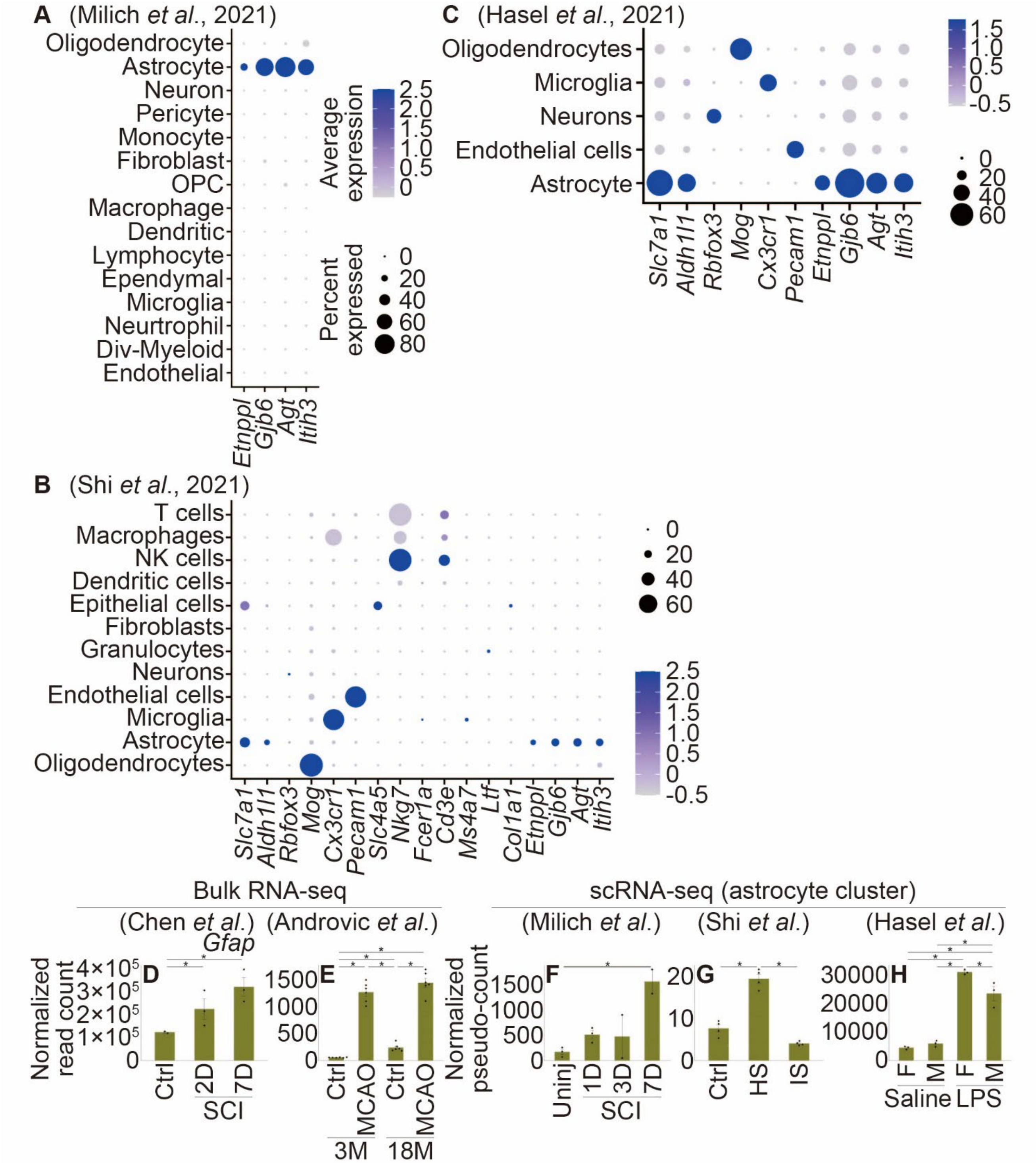

Supplementary Figure 3 Expression of cell markers and astrocytic genes in the RNA-seq database.

A-C Bubble plot of the scRNA-seq database. Blue indicates that the average expression of the genes in the cluster is high. The diameter of the bubble indicates the percentage of cells expressing the gene in the cluster. **D-H** Expression level of GFAP in bulk RNA-seq (D, E) or scRNA-seq (F-H) datasets. Vertical axes represent normalized read counts of GFAP in the tissue (D, E) or normalized pseudo-

counts in the astrocyte cluster (F-H). * $P < 0.05$, Wald test. Ctrl, control; D, days after injury; 3M, 3 months of age; Uninj, uninjured; HS, hemorrhagic stroke; IS, ischemic stroke; F, female; M, male

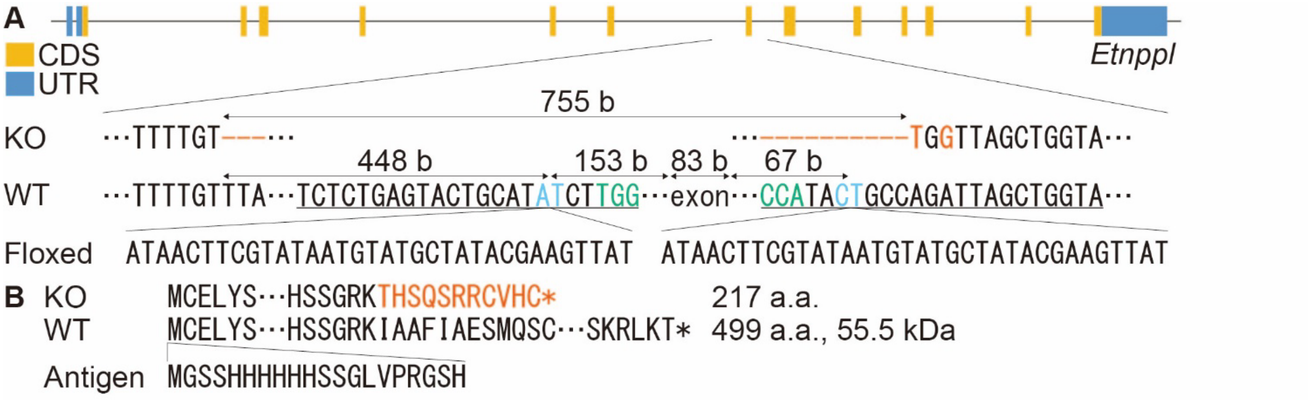

C 1st screening

#	IHC (post fixation) 0hr	6hr	1o/n	3o/n	7o/n	(FL) WB	IP
1	-	-	n.a.	n.a.	n.a.	-	n.a.
2	-	-	n.a.	n.a.	n.a.	-	n.a.
3	-	-	n.a.	n.a.	n.a.	-	n.a.
4	-	-	n.a.	n.a.	n.a.	-	n.a.
5	+	-	n.a.	n.a.	n.a.	-	n.a.
6	-	-	n.a.	n.a.	n.a.	-	n.a.
7	++	++	n.a.	n.a.	n.a.	-	+
8	-	-	n.a.	n.a.	n.a.	-	n.a.
9	-	-	n.a.	n.a.	n.a.	-	n.a.
10	-	-	n.a.	n.a.	n.a.	-	n.a.
11	-	-	n.a.	n.a.	n.a.	-	n.a.
12	-	-	n.a.	n.a.	n.a.	-	n.a.
13	-	-	n.a.	n.a.	n.a.	-	n.a.
14	++	++	n.a.	n.a.	n.a.	-	+
15	-	-	n.a.	n.a.	n.a.	-	n.a.
16	-	-	n.a.	n.a.	n.a.	-	n.a.
17	-	-	n.a.	n.a.	n.a.	-	n.a.
18	++	+	n.a.	n.a.	n.a.	-	+
19	-	-	n.a.	n.a.	n.a.	-	n.a.
20	++	+	+	-	-	-	+
21	++	+	n.a.	n.a.	n.a.	-	+
22	-	-	n.a.	n.a.	n.a.	-	n.a.
23	+	-	n.a.	n.a.	n.a.	-	n.a.
24	++	+	n.a.	n.a.	n.a.	-	+
25	+	-	n.a.	n.a.	n.a.	-	n.a.
26	-	-	n.a.	n.a.	n.a.	-	n.a.
27	-	-	n.a.	n.a.	n.a.	-	n.a.
28	+	-	n.a.	n.a.	n.a.	-	n.a.
29	++	++	n.a.	n.a.	n.a.	-	++
30	++	+	n.a.	n.a.	n.a.	-	++
31	-	-	n.a.	n.a.	n.a.	-	n.a.
32	-	-	n.a.	n.a.	n.a.	-	n.a.
33	-	-	n.a.	n.a.	n.a.	-	n.a.
34	-	-	n.a.	n.a.	n.a.	-	n.a.

#	IHC (post fixation) 0hr	6hr	1o/n	3o/n	7o/n	(FL) WB	IP
35	-	-	n.a.	n.a.	n.a.	-	n.a.
36	-	-	n.a.	n.a.	n.a.	-	n.a.
37	-	-	n.a.	n.a.	n.a.	-	n.a.
38	-	-	n.a.	n.a.	n.a.	-	n.a.
39	-	-	n.a.	n.a.	n.a.	-	n.a.
40	-	-	n.a.	n.a.	n.a.	-	n.a.
41	-	-	n.a.	n.a.	n.a.	-	n.a.
42	+	-	n.a.	n.a.	n.a.	-	n.a.
43	+	+	n.a.	n.a.	n.a.	-	+
44	-	-	n.a.	n.a.	n.a.	-	n.a.
45	-	-	n.a.	n.a.	n.a.	-	n.a.
46	++	++	++	++	++	-	++
47	+	+	n.a.	n.a.	n.a.	-	++
48	-	-	n.a.	n.a.	n.a.	-	n.a.
49	-	-	n.a.	n.a.	n.a.	-	n.a.
50	++	++	n.a.	n.a.	n.a.	-	+++
51	-	-	n.a.	n.a.	n.a.	-	n.a.
52	+	+	n.a.	n.a.	n.a.	-	+
53	-	-	n.a.	n.a.	n.a.	-	n.a.
54	-	-	n.a.	n.a.	n.a.	-	n.a.
54A	-	-	n.a.	n.a.	n.a.	-	n.a.
54B	-	-	n.a.	n.a.	n.a.	-	+
54C	-	-	n.a.	n.a.	n.a.	-	+
54A1	-	-	n.a.	n.a.	n.a.	-	+
54A2	-	-	n.a.	n.a.	n.a.	-	+
54A3	-	-	n.a.	n.a.	n.a.	-	+
55	-	-	n.a.	n.a.	n.a.	-	n.a.
56	-	-	n.a.	n.a.	n.a.	-	n.a.
57	++	+	n.a.	n.a.	n.a.	-	+
58	+	-	n.a.	n.a.	n.a.	n.a.	n.a.
59	+	-	n.a.	n.a.	n.a.	-	n.a.
60	+	-	n.a.	n.a.	n.a.	-	n.a.
61	+	-	n.a.	n.a.	n.a.	-	n.a.
62	+	-	n.a.	n.a.	n.a.	-	n.a.

#	IHC (post fixation) 0hr	6hr	1o/n	3o/n	7o/n	(FL) WB	IP
63	++	++	++	++	++	+	+++
64	-	-	n.a.	n.a.	n.a.	-	n.a.
65	-	-	n.a.	n.a.	n.a.	-	n.a.
66	-	-	n.a.	n.a.	n.a.	-	n.a.
67	+	-	n.a.	n.a.	n.a.	-	n.a.
68	-	-	n.a.	n.a.	n.a.	-	n.a.
69	+	-	n.a.	n.a.	n.a.	-	n.a.
70	+	-	n.a.	n.a.	n.a.	-	n.a.
71	+	-	n.a.	n.a.	n.a.	-	n.a.
72	-	-	n.a.	n.a.	n.a.	-	n.a.
73	+	-	n.a.	n.a.	n.a.	-	n.a.
74	++	+	n.a.	n.a.	n.a.	-	+
75	-	-	n.a.	n.a.	n.a.	-	n.a.
76	++	+	n.a.	n.a.	n.a.	-	++
77	-	-	n.a.	n.a.	n.a.	-	n.a.
78	-	-	n.a.	n.a.	n.a.	-	n.a.
79	+	-	n.a.	n.a.	n.a.	-	n.a.
80	-	-	-	-	-	-	n.a.
81	+	-	n.a.	n.a.	n.a.	-	n.a.
82	-	-	n.a.	n.a.	n.a.	-	n.a.
83	-	-	n.a.	n.a.	n.a.	-	n.a.
84	++	+	+	+	+	-	+++
85	-	-	n.a.	n.a.	n.a.	-	n.a.
86	+	-	n.a.	n.a.	n.a.	-	n.a.
87	+	-	n.a.	n.a.	n.a.	-	n.a.
88	+	-	n.a.	n.a.	n.a.	-	n.a.
89	-	-	n.a.	n.a.	n.a.	-	n.a.
90	-	-	n.a.	n.a.	n.a.	-	n.a.
91	+	-	n.a.	n.a.	n.a.	-	n.a.
92	-	-	n.a.	n.a.	n.a.	-	n.a.
93	-	-	n.a.	n.a.	n.a.	-	n.a.
94	++	+	n.a.	n.a.	n.a.	++	++
95	-	-	n.a.	n.a.	n.a.	-	n.a.

D 2nd screening

#	IHC (post fixation) 0hr	6hr	1o/n	3o/n	7o/n	(CL) WB	IP
50	n.a.	n.a.	n.a.	n.a.	n.a.	n.a.	+++
50A	++	+	+	-	-	n.a.	++++
50B	++	-	+	-	-	n.a.	++++
50C	++	n.a.	+	-	-	n.a.	+++
63	+	+	++	++	++	n.a.	n.a.
63A	++	++	++	++	++	n.a.	n.a.
63B	++	++	++	++	++	n.a.	n.a.
63C	++	++	++	++	++	n.a.	n.a.
94	n.a.	n.a.	n.a.	n.a.	n.a.	++	n.a.
94A	++	+	+	-	-	+++	n.a.
94B	++	-	-	-	-	++	n.a.
94C	++	+	+	-	-	+++	n.a.

E 3rd screening

#	IHC (post fixation) 0hr	6hr	1o/n	3o/n	7o/n	(CL) WB	IP
50A	n.a.	n.a.	n.a.	n.a.	n.a.	n.a.	++++
50A1	++	+	+	-	-	n.a.	+++
50A2	++	+	+	-	-	n.a.	+++
50A3	++	+	+	-	-	n.a.	+++
63B	++	++	++	++	++	n.a.	n.a.
63B1	++	++	++	++	++	n.a.	n.a.
63B2	++	++	++	++	++	n.a.	n.a.
63B3	++	++	++	++	++	n.a.	n.a.
94A	n.a.	n.a.	n.a.	n.a.	n.a.	+++	n.a.
94A1	++	+	+	-	-	++	n.a.
94A2	++	+	+	-	-	++	n.a.
94A3	++	+	+	-	-	++	n.a.

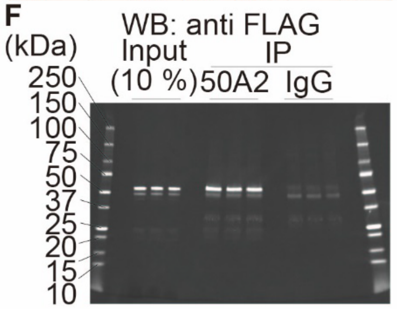

Supplementary Figure 4 Screening of monoclonal antibodies.

A Genomic structure and sequence of *Etnppl* in WT and KO mice. Line indicates intron, yellow boxes indicate coding sequence (CDS), blue boxes indicate untranslated region (UTR). The gRNA target sequences with PAM (green) are underlined. In floxed mice, loxP sequences are inserted between the bases indicated in blue. Hyphens indicate deletions. Orange characters indicate a sequence different

from that of the WT. **B** Amino acid sequence of ETNPPL. * Stop codon. a.a., amino acid. The sequence of the antigen used to produce anti-ETNPPL monoclonal antibodies (recombinant His-tagged ETNPPL) is also shown. **C-E** Summary of monoclonal antibody clone screening. The screening experiments were not repeated. o/n, overnight; n.a., not analyzed; FL, fluorescence; CL, chemiluminescence. **F** Representative image of WB using IP samples. The input was the lysate of FLAG-tagged ETNPPL expressing cells. IP was performed using anti- ETNPPL clone 50A2 or normal rat IgG. WB was performed using anti-FLAG antibody. Input was 10 % of IP sample. Three samples in each group were technical replicates (all inputs were aliquots from the same tube). The lanes between each group or marker are empty lanes.

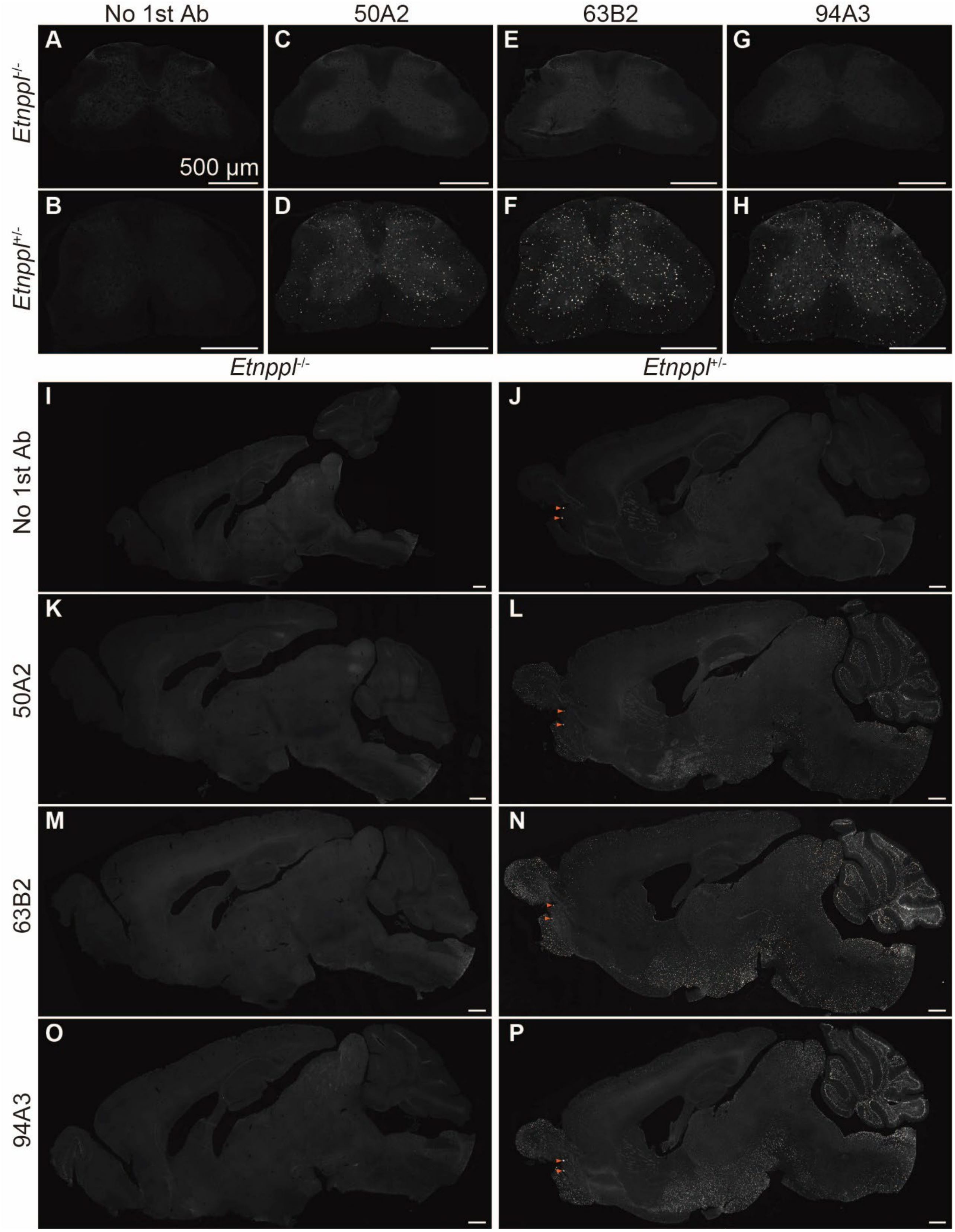

Supplementary Figure 5 Image of whole brain of validation of anti-ETNPPL monoclonal antibodies.

Coronal section of the cervical cord (A-H) or parasagittal section of the whole brain (I-P) of 8 W female *Etnppl*$^{-/-}$ (A, C, E, G, I, K, M, O) or *Etnppl*$^{+/-}$ (B, D, F, H, J, L, N, P) mice are shown. Anti-ETNPPL monoclonal antibody clones 50A2 (C, D, K, L), 63B2 (E, F, M, N), or 94A3 (G, H, O, P) were used as primary antibody, or no primary antibody was applied (A, B, I, J). Orange arrowheads indicate autofluorescence, which was also observed in the no-primary-antibody group. Scale bars: 500 μm.

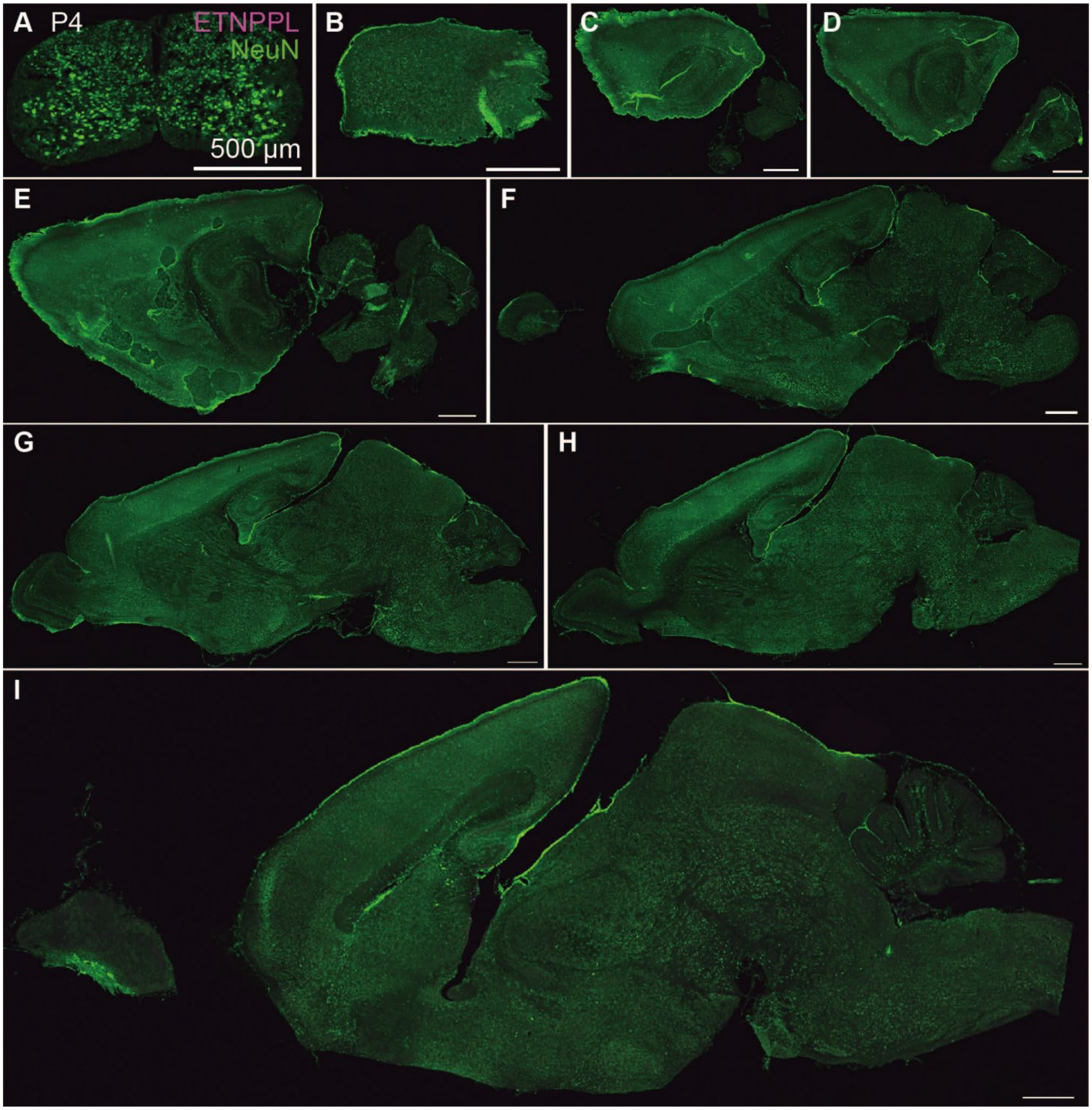

Supplementary Figure 6 Localization of ETNPPL in the whole brain of P4 mice with anti-NeuN staining.

Images in Fig.4 are presented as merged images of ETNPPL (magenta) and NeuN (green) to clarify the anatomical location.

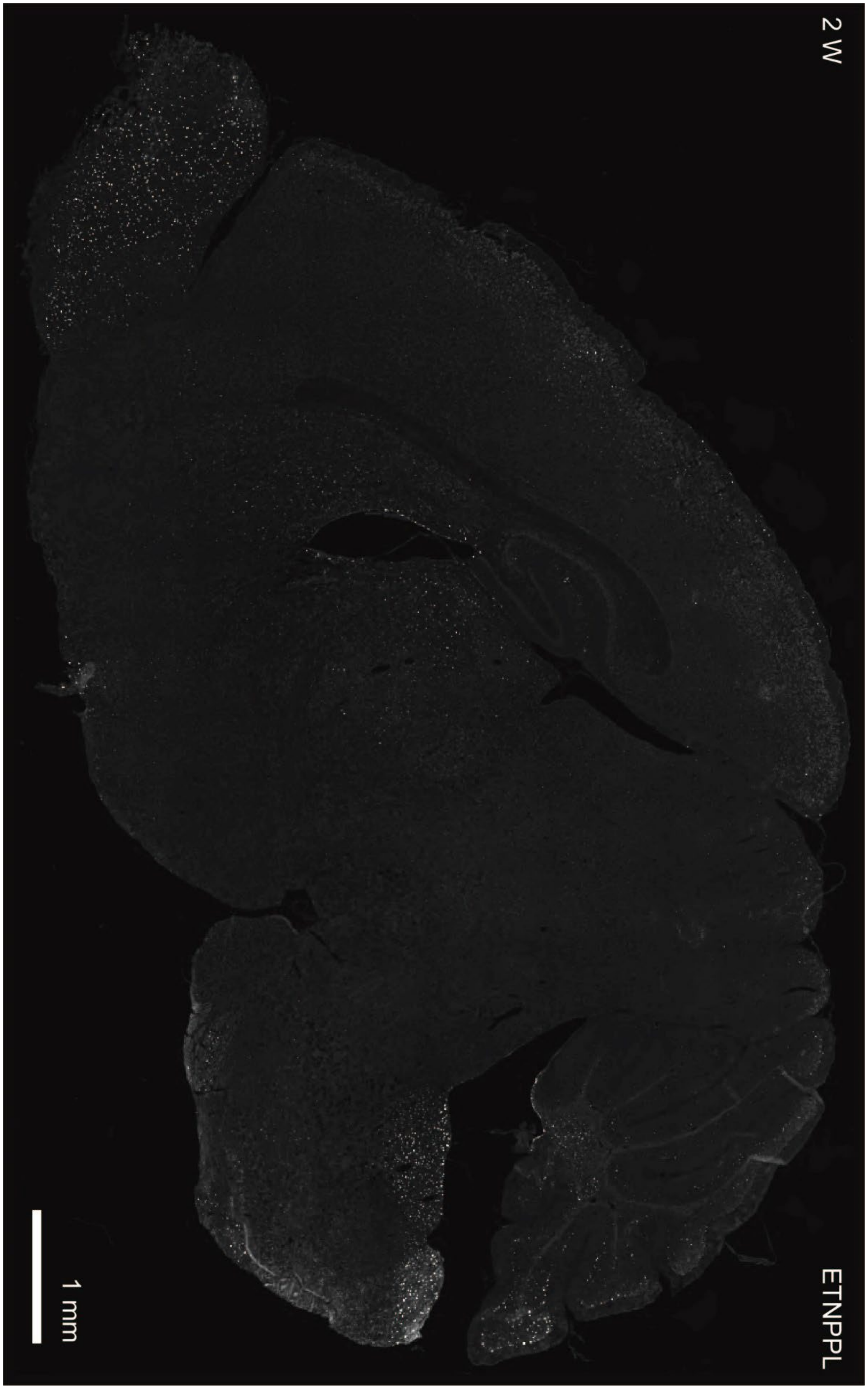

Supplementary Figure 7 High quality image of ETNPPL in the whole brain of 2 W mice.

A high quality image of Fig. 5O is shown. Scale bar: 1 mm.

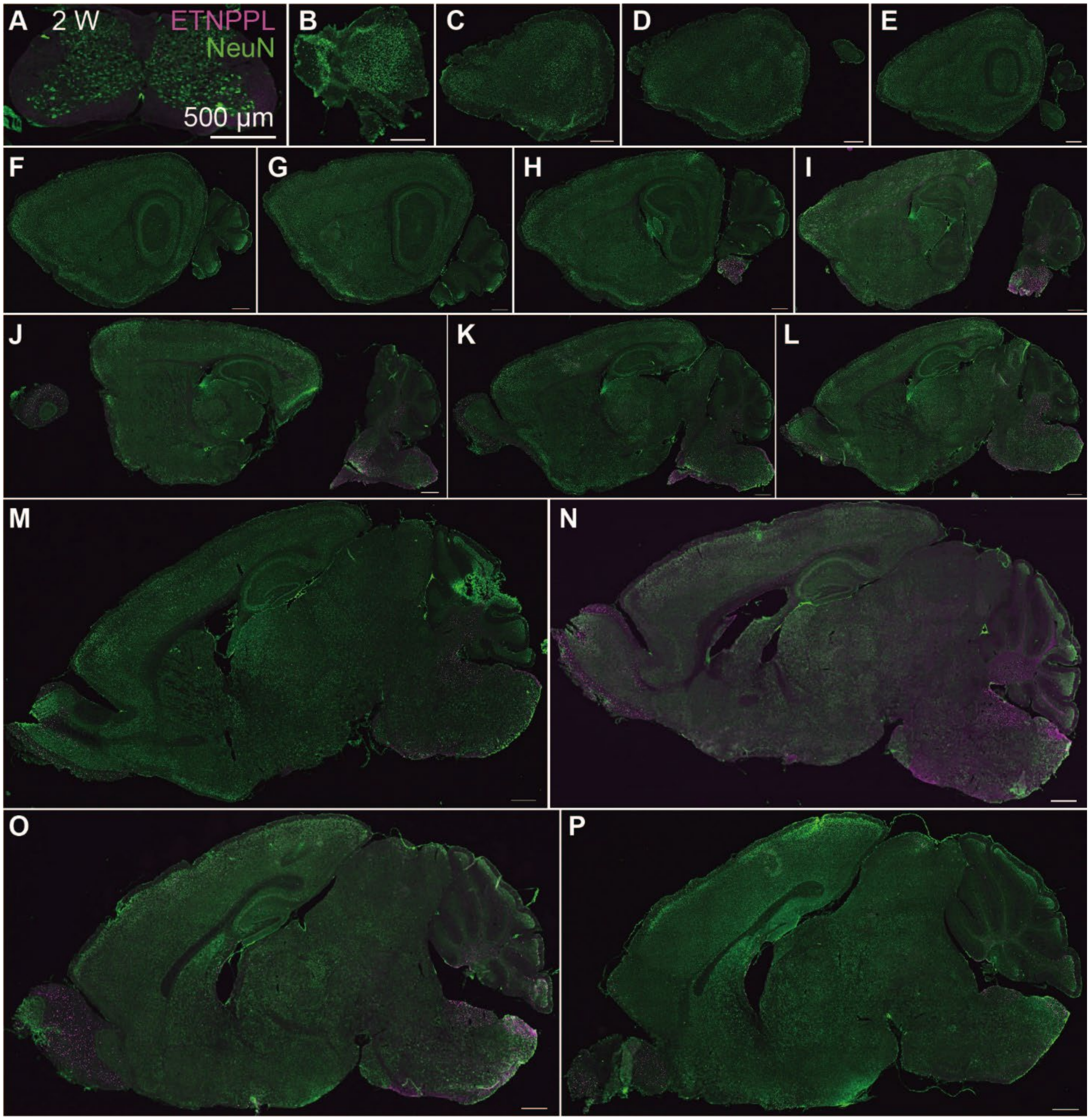

Supplementary Figure 8 Localization of ETNPPL in the whole brain of 2 W mice with anti-NeuN staining.

Images in Fig.5 are presented as merged images of ETNPPL (magenta) and NeuN (green) to clarify the anatomical location.

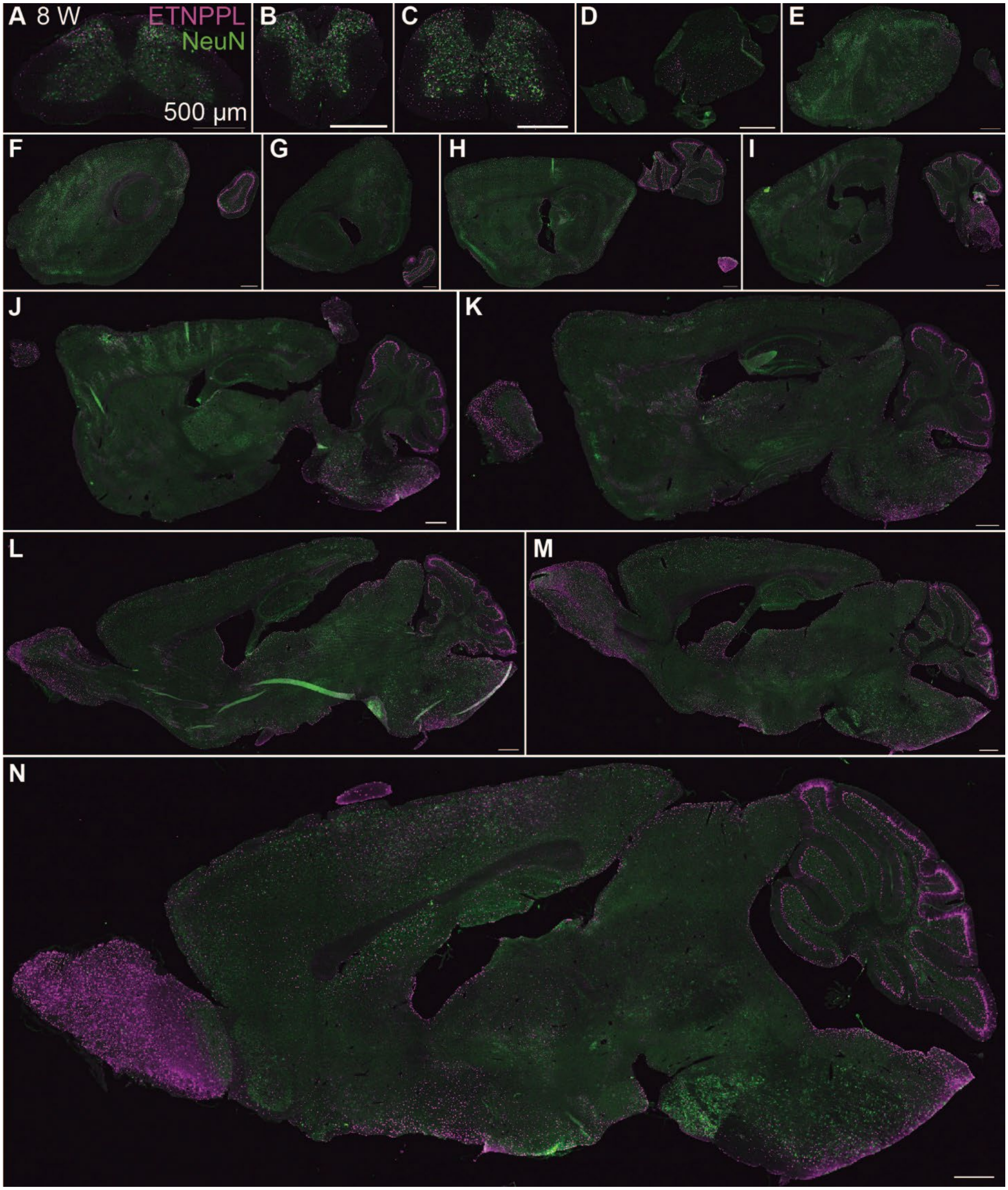

Supplementary Figure 9 Localization of ETNPPL in the whole brain of 8 W mice with anti-NeuN staining

Images in Fig.6 are presented as merged images of ETNPPL (magenta) and NeuN (green) to clarify the anatomical location.

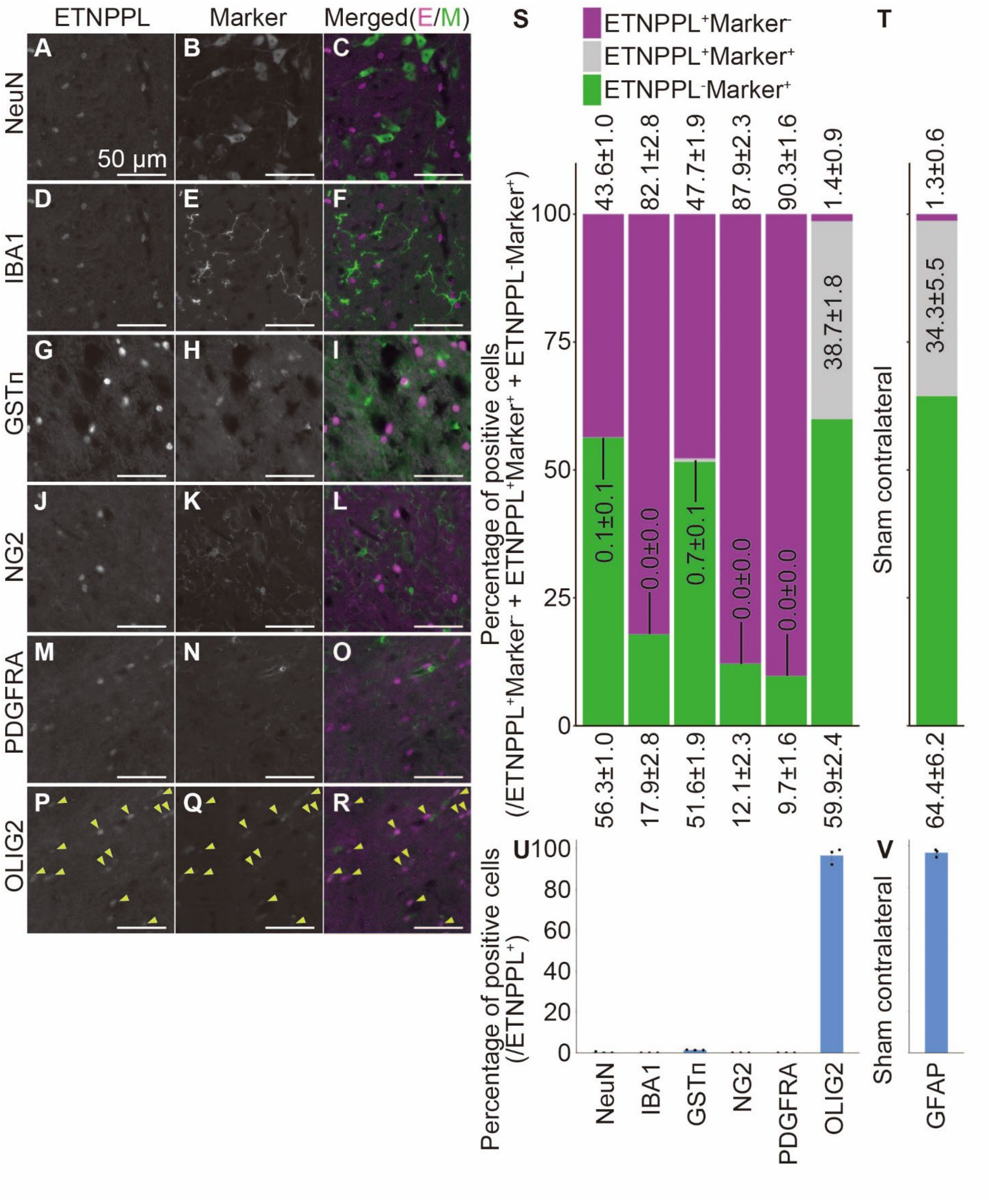

Supplementary Figure 10 Cellular specificity of ETNPPL in the cervical cord of adult mice.

A-R Representative images of multiple staining of ETNPPL and cell markers in the gray matter of the cervical cord of 8 W female mice. Signals for ETNPPL (A, D, G, J, M, P), NeuN (B), IBA1 (E), GSTπ (H), NG2 (K), PDGFRA (N), or OLIG2 (Q) and merged images (C, F, I, L, O, R) are shown. In the

merged images, magenta indicates the signal for ETNPPL, and green indicates the signal for cell markers. Yellow arrowheads indicate cells double-positive for ETNPPL and OLIG2. Some experiments were not performed on the same day, and the brightness of the ETNPPL channels was adjusted differently. Scale bars: 50 µm. The same ETNPPL channel images were used in (A), (C), (D), and (F), or (M), (O), (P), and (R). **S-V** Quantification of the percentage of (S, T) cells expressing ETNPPL and cell markers or (U, V) $ETNPPL^+$ cells labeled with a certain cell-type specific marker. The right side of intact mice (S, U, shown in this figure) or the contralateral (right) side of sham mice (T, V, shown in Fig. 9) were used. Magenta indicates ETNPPL single positive cells, green indicates cell marker single positive cells, and gray indicates cells that are double positive for ETNPPL and cell markers. Mean ± S.E.M. n = 3 individuals, each of which is the average of four different sections.

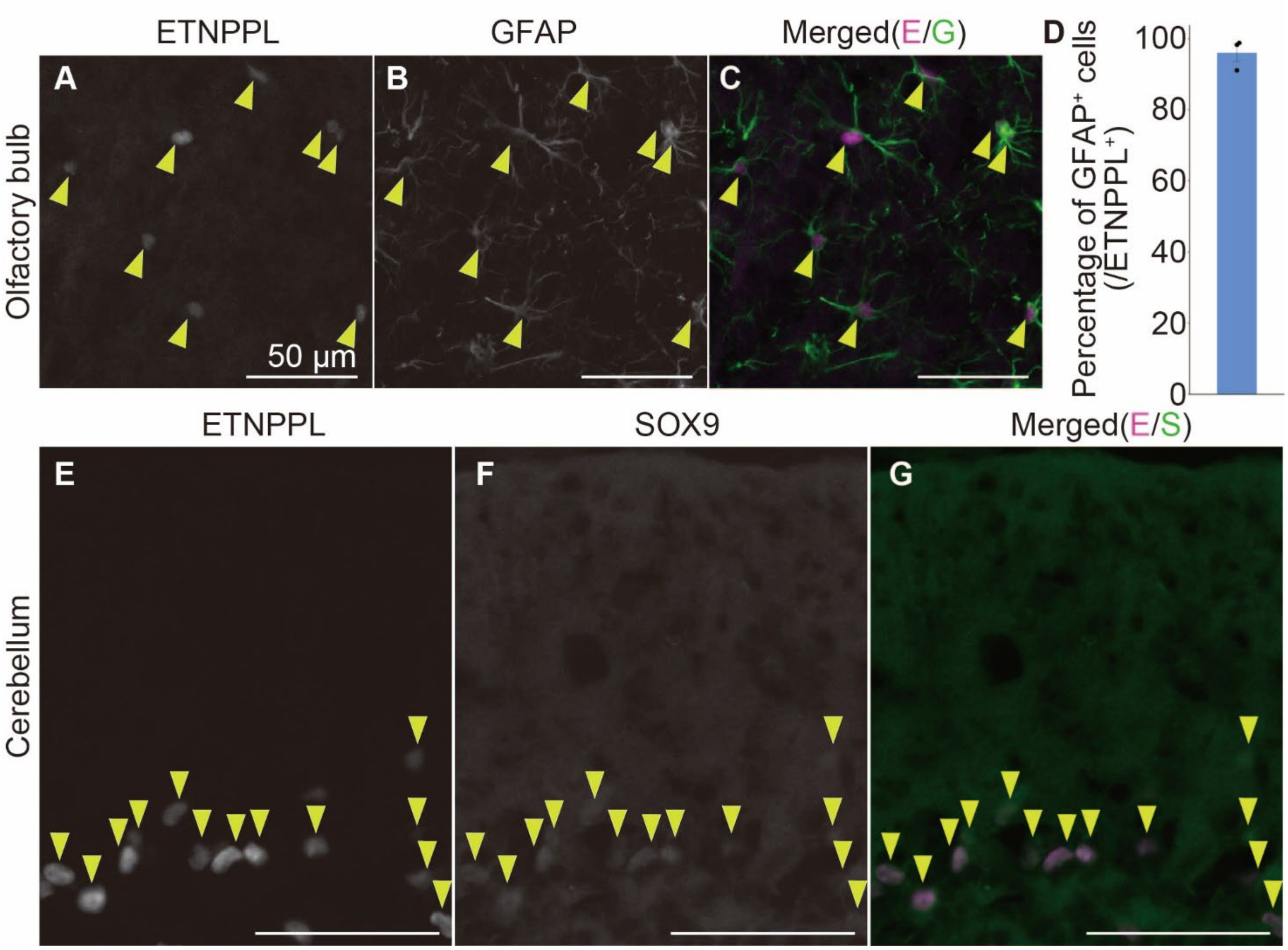

Supplementary Figure 11 Expression of ETNPPL and astrocytic markers in olfactory bulb and cerebellum.

A-C Representative images of multiple staining of ETNPPL and GFAP in the olfactory bulb of 8 W female mice. Signal for ETNPPL (A), GFAP (B) and a merged image (C) are shown. Magenta indicates signal for ETNPPL and green indicates signal for GFAP. **D** Quantification of the percentage of ETNPPL$^+$ cells labeled with GFAP. Mean ± S.E.M. n = 3 individuals, each of which is the average of three different sections. **E-G** Representative images of multiple staining of ETNPPL and SOX9 in the cerebellum of 8 W female mice. Signal for ETNPPL (E), SOX9 (F) and a merged image (G) are shown. Magenta indicates signal for ETNPPL and green indicates signal for SOX9. Yellow arrowheads indicate cells double-positive for ETNPPL and GFAP (A-C) or SOX9 (E-G).

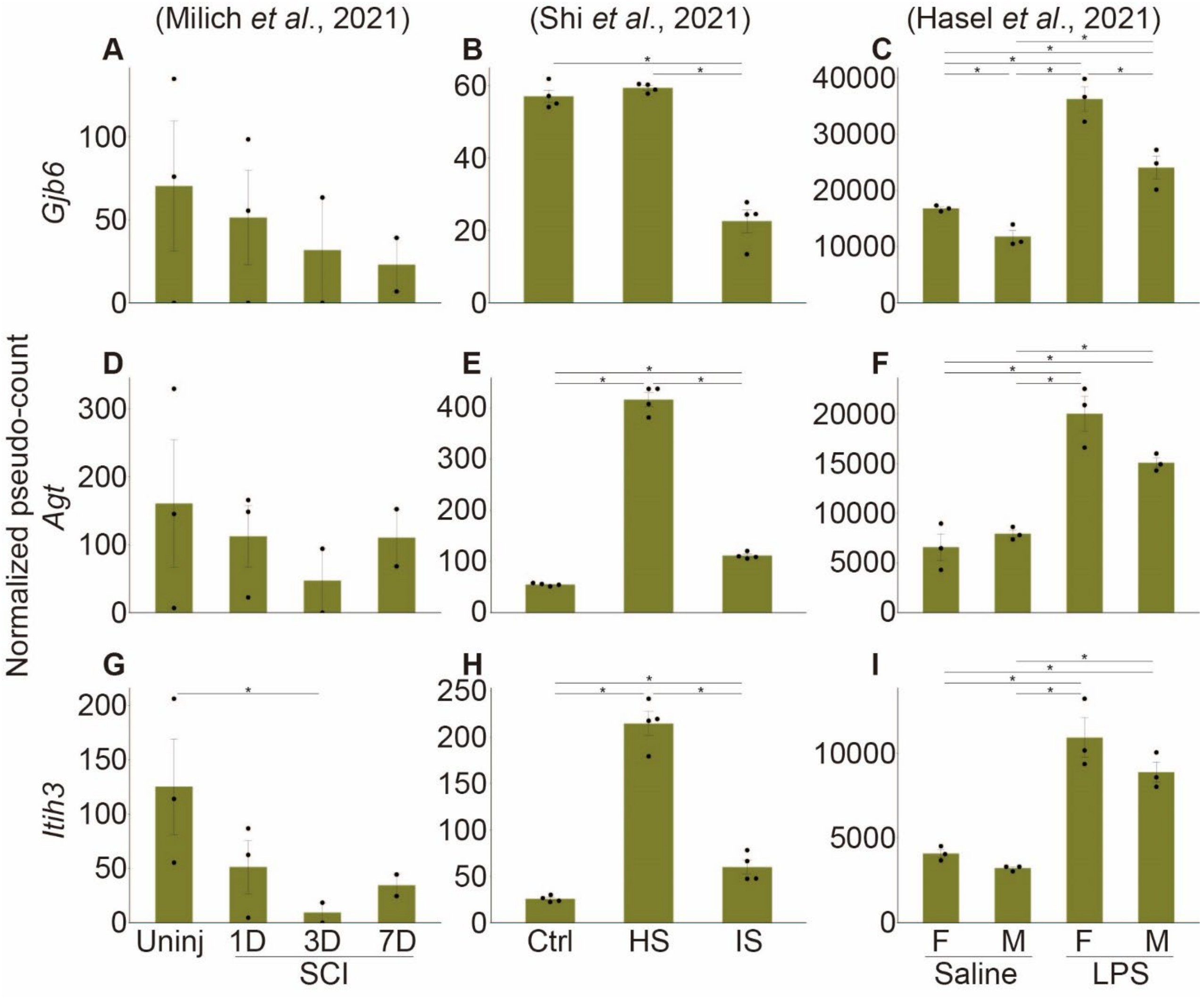

Supplementary Figure 12 Expression change of astrocyte subtype genes in several diseases in database.

Vertical axes represent normalized pseudo-counts of *Gjb6* (A-C), *Agt* (D-F), or *Itih3* (G-I) in the astrocyte cluster. * $P < 0.05$, Wald test. Ctrl, control; D, days after injury; Uninj, uninjured; HS, hemorrhagic stroke; IS, ischemic stroke; F, female; M, male.

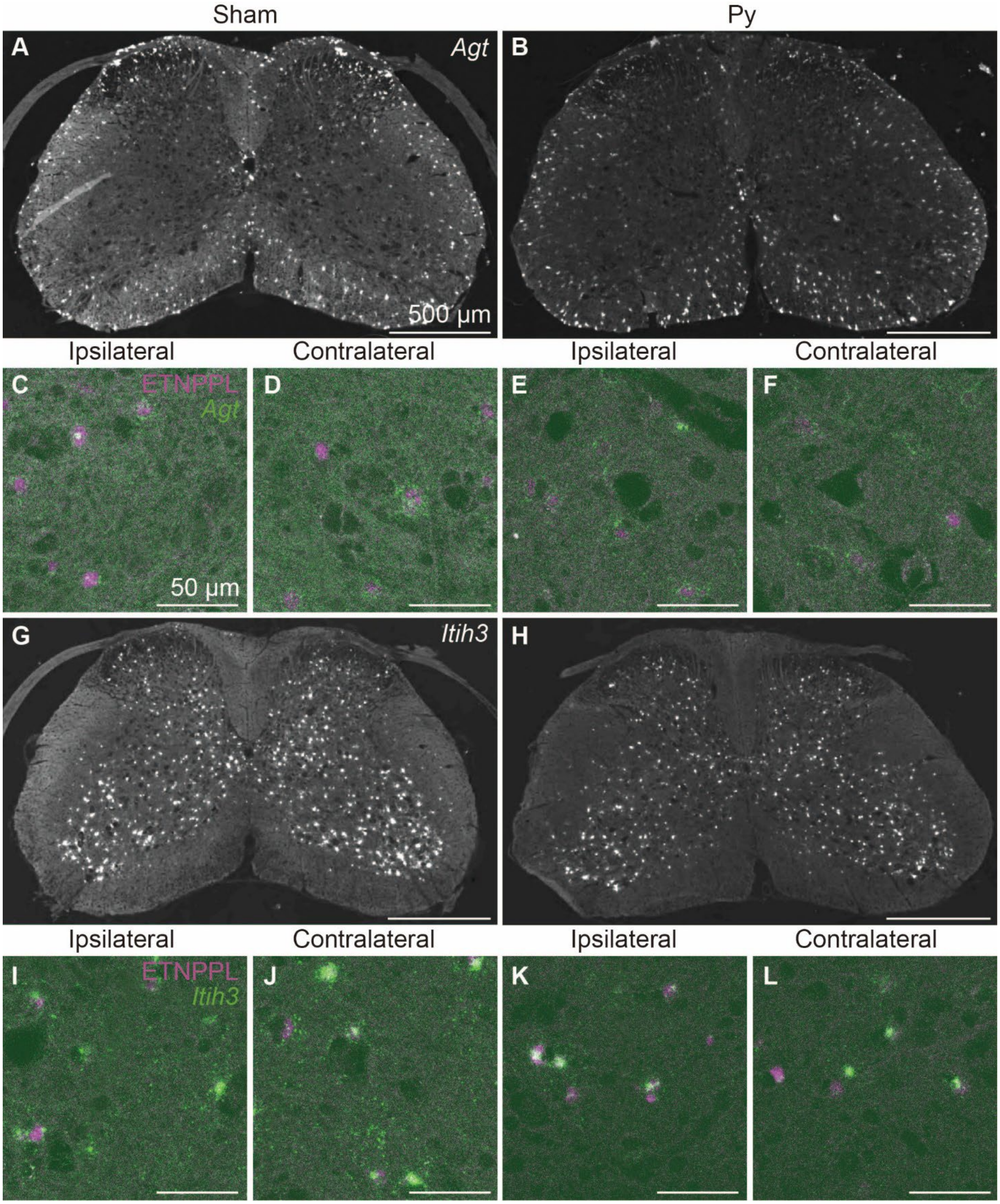

Supplementary Figure 13 Localization of *Agt* or *Itih3*.

Representative images of coronal section (A, B, G, H), or enlarged confocal images of ipsilateral (C, E, I, K) or contralateral side (D, F, J, L) of the gray matter of the cervical cord of 8 W female mice in sham (A, C, D, G, I, J) or pyramidotomy groups (B, E, F, H, K, L). Signals for *Agt* (A, B) and *Itih3* (G,

H), merged images of ETNPPL (magenta) and *Agt* (green, C-F), and merged images of ETNPPL (magenta) and *Itih3* (green, I-L) are shown. Scale bars:500 µm (A, B, G, H) or 50 µm (C-F, I-L).

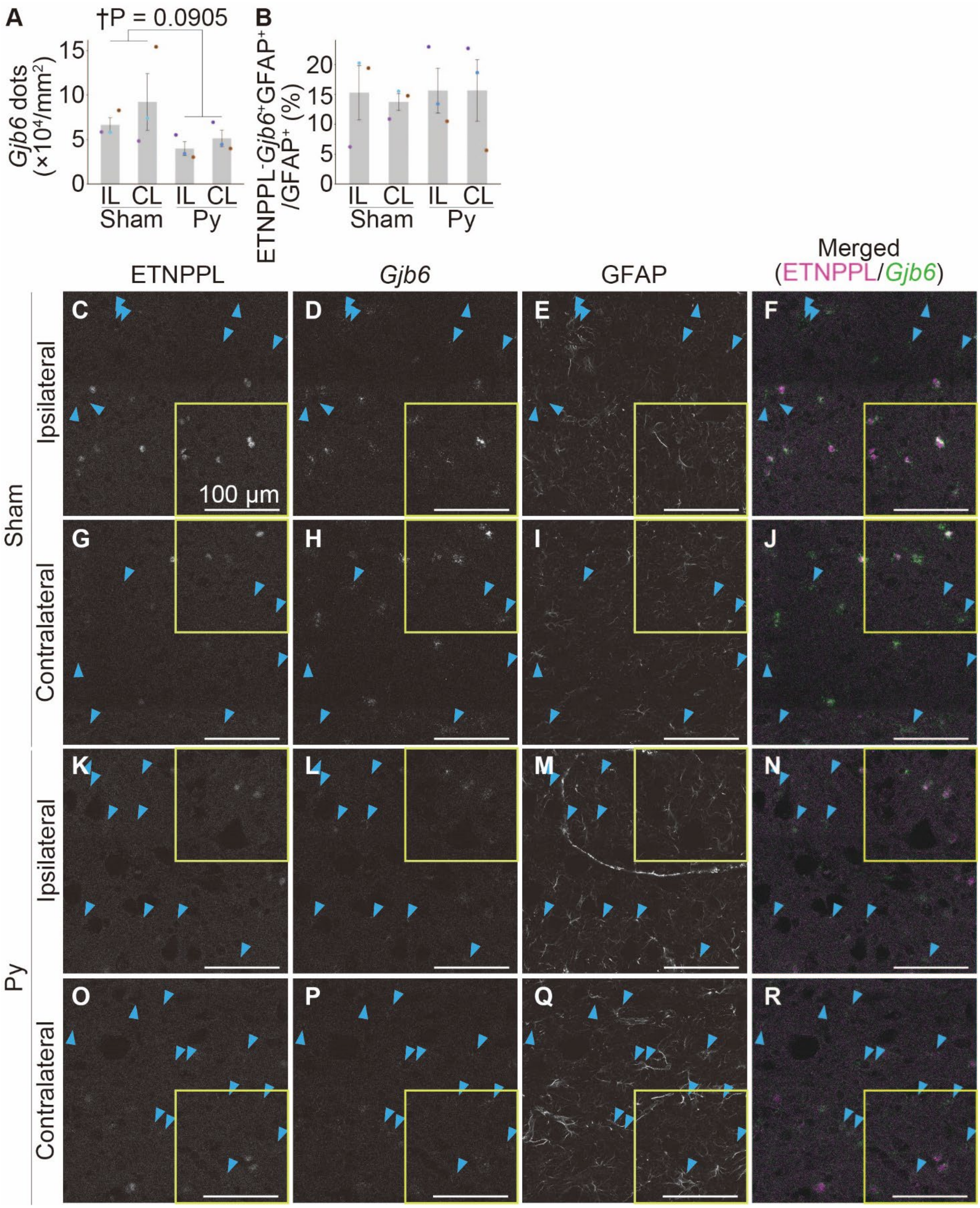

Supplementary Figure 14 Further quantification of *Gjb6*.

A, B Quantification of changes after pyramidotomy. The vertical axes represent the number of dots for *Gjb6*-signal (A) or the percentage of ETNPPL$^-$*Gjb6*$^+$GFAP$^+$ cells to total GFAP$^+$ astrocytes (B). Mean

± S. E. M. n = 3 individuals, each of which is the average of four different sections. The same color indicates the same individual. IL, ipsilateral; CL, contralateral. †Sham VS Py, two-way ANOVA. **C-R** Wider images of Fig. 10C-R. The gray matter of the ipsilateral (C-F, K-N) or contralateral side (G-J, O-R) of the sham (C-J) or pyramidotomy group (K-R) are shown. Signals for ETNPPL (C, G, K, O), *Gjb6* (D, H, L, P), or retrieved GFAP (E, I, M, Q) are shown. In merged images (F, J, N, R), magenta indicates ETNPPL and green indicates *Gjb6*. Blue arrowheads indicate the $ETNPPL^{-}$*Gjb6*$^{+}$$GFAP^{+}$ cells. Scale bars: 100 µm. Yellow boxes indicate area magnified in Fig. 10C-R.
